# A Controlled Study of Physics-Informed Auxiliary Supervision and Scalar Triplet Attention in Equivariant Molecular Force Fields

**DOI:** 10.3390/molecules31121987

**Published:** 2026-06-06

**Authors:** Chenglei Han, Fei Wang, Jiyao Liang, Jie Cui, Lin Li

**Affiliations:** 1 School of Integrated Circuits, Shandong University, Jinan 250101, China; chengleihan@mail.sdu.edu.cn (C.H.); jiyaol@mail.sdu.edu.cn (J.L.); 2 School of Electronic Science and Engineering, Xiamen University, Xiamen 361100, China; cj123456789cj02@163.com (J.C.); lilin@xmu.edu.cn (L.L.)

**Keywords:** equivariant graph neural network, machine learning interatomic potential, auxiliary geometric supervision, many-body features, rMD17

## Abstract

Machine-learned molecular force fields require many-body geometry, but obtaining it through Clebsch–Gordan tensor products is computationally expensive. For a strong no-Clebsch–Gordan backbone such as GotenNet, we ask whether the limitation in handling three-body geometry is one of representational capacity or one of training supervision, and separate the two factors with three controlled probes on a single-seed, paper-aligned rMD17 aspirin split. (i) While frame projection of tensor features is comparable to scalar cos-angle triplet cross-attention (SCTA) at pilot scale, algebraically its diagonal scalar collapses to a frame-independent inner product and the remaining channel is parity-odd, making SCTA’s cos-angle input the principled O(3) scalar choice. (ii) SCTA matches GotenNet’s converged force accuracy within ∼0.4% without independent gain, indicating that three-body representational capacity is not the binding constraint. (iii) A graph-level auxiliary loss on bond-angle and dihedral statistics gives the best force mean absolute error (MAE; 0.1280 vs. 0.1303 kcal/mol/Å) and reduces epochs-to-validation-target by 26–55%. Cross-molecule probes do not extend this finding; a paired salicylic acid comparison shows a directional degradation that, under a configuration-level paired block bootstrap, is significant and opposite in sign to the aspirin effect. Across three random seeds, the auxiliary force-MAE gain is small and seed-dependent but consistently reduces seed-to-seed variance and accelerates convergence, indicating that low-cost three-body supervision can be a more effective lever than added three-body capacity.

## 1. Introduction

Accurate prediction of molecular potential energies and atomic forces from 3D atomic coordinates is fundamental to computational chemistry, enabling molecular dynamics, conformational sampling, and modeling of chemical and material processes [1,2,3]. Density functional theory (DFT) is widely used to generate reference energies and forces for small- and medium-sized molecular systems, but its rapidly increasing cost with system size limits routine use in long trajectories or large molecular ensembles. Machine learning of interatomic potentials offer an attractive alternative. Once trained on reliable reference data, such models can approximate DFT-level energy and force evaluations at much lower inference cost [4,5,6,7,8,9,10]. Nevertheless, the cost of generating reference data and training stable force models makes sample-efficient learning a central requirement for practical molecular force-field development.

Among ML interatomic potentials, equivariant graph neural networks have become a leading approach for molecular energy and force prediction [11,12,13,14,15,16,17]. Their appeal comes from encoding the symmetries of molecular physics directly in the model: predicted energies should be invariant to translations and rotations of the coordinate frame, whereas predicted forces should transform equivariantly with the atoms. Beyond pairwise distances, however, accurate molecular force fields also depend on angular and higher-body geometry. Distance-based representations can fail to distinguish configurations that differ by angular rearrangement [18]. Therefore, many successful molecular models incorporate three-body or higher-order information through radial-angular basis functions, tensor features, spherical harmonics, equivariant attention, or vector–scalar interaction blocks [19,20,21,22,23,24,25,26,27,28,29]. This leads to a central design question for practical force-field learning, namely, how can a model expose useful angular many-body information while keeping training stable and computationally affordable?

These developments expose a practical tension in equivariant force-field design. Scalar-invariant models are usually efficient and stable to train, but their access to molecular geometry depends on the invariant radial, angular, or higher-body quantities supplied to the network; related expressivity limits have been analyzed for both graph and invariant geometric models [30,31]. Tensor-based models provide a systematic route to richer equivariant representations by propagating spherical or tensor features [32], but their cost grows steeply with angular order and channel width, which constrains depth, model size, and routine hyperparameter exploration under fixed compute budgets [33]. In practice, many high-performing models rely on low-order truncations or carefully engineered geometric channels rather than on unconstrained higher-order tensor representations. This motivates a more targeted question for small-molecule force-field learning: can useful three-body geometric information be exposed to an equivariant backbone through lightweight scalar mechanisms without the need to rely on Clebsch–Gordan tensor products?

Recent work has sought to reduce or avoid explicit Clebsch–Gordan (CG) tensor products while retaining strong equivariant-model accuracy [34]. EST [35] uses attention in a spherical spatial domain, Graph ACE [36] provides a cluster-expansion view of equivariant message passing, Geodite [37] removes CG tensor products from a GotenNet-style backbone using high-degree inner products and physical priors, and MARA [38] introduces continuous SE(3)-equivariant spherical attention as an efficient approximation to equivariant interactions without high-order tensor products. From a different angle, HEGNN [33] shows that increasing the steerable-feature degree (within a no-CG scalarization scheme) can recover angular information without expensive tensor coupling. This finding is complementary to our own, since if intrinsic high-degree capacity is already adequate, then supervision becomes the dominant limiting factor. Beyond no-CG molecular force-field designs, the broader literature offers several adjacent points of comparison. Triplet Graph Transformer (TGT) [39] introduces triplet attention into a 2D molecular graph transformer with an auxiliary interatomic distance prediction stage, demonstrating that explicit three-body channels improve property prediction on graph-level benchmarks (PCQM4Mv2, QM9). Scalarization-compatible Triplet Cross-Attention (SCTA) is used; it can be viewed as the equivariant 3D-scalar instance of the same intuition, except evaluated for its incremental value over an already-competent equivariant backbone rather than as a standalone architecture. Several recent equivariant designs avoid or restructure the CG transform: FreeCG [40] performs CG on permutation-invariant abstract edges to widen the CG design space, E2Former [41] uses a linear-scaling tensor-product attention via Wigner-6j convolution, GeoMFormer [42] couples invariant and equivariant transformer streams via cross-attention, HotPP [43] performs E(*n*)-equivariant Cartesian-tensor message passing for high-order outputs (dipole, polarizability), and TACE [44] provides a unified irreducible Cartesian-tensor framework. Similarly, CEITNet [45] performs many-body coupling in Cartesian channel space and targets high-order crystal tensors. These designs are largely complementary to ours; they expand or restructure architectural capacity for many-body and tensor information, whereas we ask whether adding three-body supervision is a more effective lever at fixed backbone capacity (GotenNet) than adding further three-body capacity. We do not benchmark against these models directly because their targets and design budgets differ, but do situate them in the design space when interpreting our findings. Existing studies in this area mainly target architectural expressiveness or inference efficiency. We ask a narrower experimentally controlled question for small-molecule force-field training: when added to a strong no-CG backbone, do lightweight scalar geometric priors change convergence or final accuracy, and where do their benefits fail to transfer?

We use GotenNet [46] as this backbone because it achieves competitive revised MD17 (rMD17) [47] accuracy without explicit CG tensor products while implicitly encoding angular information through hierarchical tensor refinement and equivariant feed-forward updates. Beyond comparing add-on mechanisms, we ask whether the backbone’s treatment of three-body geometry is limited by representational capacity, or by training supervision. We separate the two factors with three controlled probes. Two of them add representational capacity: the frame projection probe projects tensor features onto a triplet-local frame, while SCTA provides a zero-initialized residual branch that attends over neighbor pairs using scalar features and a cosine-angle basis. The third probe adds supervision rather than capacity in the form of a graph-level auxiliary loss on bond-angle and dihedral statistics. The main experiments form a controlled single-seed ablation on rMD17 aspirin; limited ethanol, uracil, and salicylic acid probes are reported separately to delimit the aspirin findings rather than to generalize them.

Our main contributions are as follows:

- **Capacity vs. supervision.** We frame the problem of adding three-body geometry to a no-Clebsch–Gordan backbone as a choice between representational capacity and training supervision, and separate the two using three controlled probes on rMD17 aspirin. The main finding is that added three-body capacity does not move converged accuracy, whereas low-cost three-body supervision does.
- **Frame projection vs. SCTA.** We establish two structural properties of a frame-projection scalar branch: the diagonal frame-projected pairwise feature collapses exactly to a frame-independent tensor inner product, and the only genuinely frame-dependent channel is a parity-odd pseudoscalar. At pilot scale the two branches achieve comparable validation mean absolute error (MAE), so we present the analysis as a design rationale: SCTA’s cos-angle input is a true O(3)-invariant three-body scalar that requires no frame construction.
- **SCTA as a capacity control.** A correctly designed scalar triplet branch with complexity O(|T|·D) and a cosine-angle basis matches GotenNet’s converged force MAE within ∼0.4% on aspirin, but yields no robust independent gain. We interpret this neutral result as evidence that the GotenNet backbone’s implicit angular pathway already supplies the relevant three-body capacity, so adding more representational capacity is not the binding constraint.
- **Auxiliary supervision as the effective lever.** A graph-level auxiliary loss on bond-angle and dihedral statistics, which adds three-body supervision rather than capacity, gives the best force MAE in our ablation (0.1303→0.1280 kcal/mol/Å) while preserving energy accuracy and reducing the epochs to selected validation targets by 26–55%. Limited ethanol, uracil, and salicylic acid probes serve only to delimit the scope of this finding at single-seed precision, and are not put forward as a molecule-independent claim.

## 2. Results and Discussion

### 2.1. Main Results on rMD17 Aspirin

We first compare the reproduced vanilla GotenNet baseline, GotenNet with SCTA, and their auxiliary-supervised variants on the aspirin split of rMD17. All in-house runs use the paper-aligned configuration ($d_{ne}=192$ hidden channels, 16 interaction layers, $L_{\max}=2$ steerable features), 3000 training epochs, AdamW, and a fixed random seed. Table 1 reports test set MAE on the held-out 1000-configuration test split, using kcal/mol for energy and kcal/mol/Å for force.

The force column provides the clearest outcome. Auxiliary-supervised variants form a narrow 0.1280–0.1292 kcal/mol/Å band, improving over the corresponding no-auxiliary runs at 0.1298–0.1303 kcal/mol/Å. The lowest force MAE in our ablation is obtained by GotenNet + aux (physics) at 0.1280 kcal/mol/Å, compared with 0.1303 kcal/mol/Å for reproduced vanilla GotenNet. By contrast, adding the SCTA capacity branch changes converged force MAE by only about 1% at the fixed auxiliary setting. Therefore, the auxiliary geometric supervision is associated with a small but directionally favorable shift in the converged force number, whereas the added three-body representational capacity of SCTA is not. At single-seed precision, the 0.0023 kcal/mol/Å gap (1.8%) should not on its own be treated as a statistically established effect; the basis for our conclusion is the consistent pattern across the six controlled configurations rather than any single pairwise difference.

The energy column highlights a separate tradeoff in which GotenNet + aux (physics) preserves the low energy MAE of the vanilla GotenNet baseline (0.0357 vs. 0.0353 kcal/mol), whereas SCTA + aux (hybrid) increases energy MAE to 0.0538 kcal/mol. We attribute this to an interaction between the SCTA residual branch and the hybrid auxiliary target rather than to a failure of auxiliary supervision in general. Because all in-house aspirin comparisons are single-seed results on one molecule, the narrow ranking within the 0.1280–0.1292 kcal/mol/Å force band should not be over-interpreted; the more robust conclusions are the component-level pattern analyzed below and the transfer behavior reported in Section 2.5.

#### Analytic Test Set Confidence Intervals

To place a finite-sample uncertainty around the point estimates in Table 1, we compute analytic Wald-type 95% confidence intervals on each test MAE, under the assumption that residuals are approximately independent across the 1000 test configurations for energy (n=1000) and across all force components (*n* = 63,000 for 21 atoms × 3 components × 1000 configurations): $\mathrm{SE}\left(\mathrm{MAE}\right)=\sqrt{(\mathrm{MSE}-{\mathrm{MAE}}^{2})/n}$. This is a test set CI, not a seed-to-seed CI; it captures finite-sample variation under a fixed model and an independence assumption that may understate the true error if within-trajectory residuals are correlated. A block bootstrap over trajectory segments more faithfully reflects the temporal structure of the rMD17 test split. We recover the per-configuration residuals from the released best-validation checkpoints and report such a configuration-level paired block bootstrap in the analyses below. With the above caveat, the resulting analytic intervals are (energy in kcal/mol, force in kcal/mol/Å):

- GotenNet (reproduced): E=0.0353[0.0329,0.0378], F=0.1303[0.1290,0.1316]
- SCTA: E=0.0360[0.0337,0.0383], F=0.1298[0.1285,0.1311]
- GotenNet + aux (hybrid): E=0.0370[0.0345,0.0394], F=0.1292[0.1279,0.1305]
- GotenNet + aux (physics): E=0.0357[0.0333,0.0381], F=0.1280[0.1267,0.1293]
- SCTA + aux (hybrid): E=0.0538[0.0512,0.0565], F=0.1287[0.1274,0.1300]
- SCTA + aux (physics): E=0.0397[0.0373,0.0421], F=0.1290[0.1277,0.1303]

On force, the 0.0023 kcal/mol/Å gap between GotenNet (0.1303) and GotenNet + aux (physics) (0.1280) corresponds to a two-sample *z*-statistic of ≈2.5 (using ${\mathrm{SE}}_{\Delta }=\sqrt{{\mathrm{SE}}_{1}^{2}+{\mathrm{SE}}_{2}^{2}}\approx 9.2\times 10^{-4}$ under independence), and the two 95% intervals overlap only at their boundary. However, this analytic interval treats the 63,000 force components as independent, and as such is optimistic. A configuration-level paired block bootstrap that resamples the ∼1000 correlated test configurations yields intervals roughly three times wider, under which the single-seed force gap is no longer distinguishable from test set sampling noise. The seed-level robustness of the effect is examined in Section 2.2. On energy, the intervals are wider (±0.0024 kcal/mol for vanilla GotenNet) and the GotenNet vs. GotenNet + aux (physics) intervals overlap substantially, consistent with the paper-level claim that GotenNet + aux (physics) *preserves* energy accuracy rather than improving it. The same caveat as in the previous paragraph applies: this is finite-test-sample uncertainty, not seed-to-seed uncertainty, and the GotenNet reference values cited in Table 1 are five-split averages reported in [46] without per-split variance, so a direct seed-level comparison is not available.

### 2.2. Multi-Seed Validation on Aspirin

The aspirin comparison above is single-seed. To test whether the auxiliary force-MAE improvement is robust to initialization, we retrained vanilla GotenNet and GotenNet + aux (physics) under three random seeds on the same paper-aligned split (splits_0) and training protocol, then evaluated each model at its best-validation checkpoint on the held-out test set. Table 2 reports the test MAE as mean ± standard deviation over the three seeds.

The auxiliary loss lowers the mean force MAE (0.1289→0.1274 kcal/mol/Å); more robustly, it reduces the seed-to-seed standard deviation roughly threefold (0.0018→0.0006). A configuration-level paired block bootstrap of the force residuals makes the per-seed difference significant on seed 2 (Δ=0.0020 kcal/mol/Å, p<0.001) but not on seed 3 (Δ=0.0001, p=0.87), where the vanilla baseline itself converges to its lowest force MAE; therefore, the size of the effect is comparable to the baseline’s own seed-to-seed variability. Accordingly, we read the auxiliary supervision as a modest variance-reducing lever, not as a substantial gain in converged accuracy, and do not over-interpret the single-seed force band of Section 2.1. Energy accuracy is preserved, with both models reaching a test energy MAE of about 0.0355 kcal/mol across seeds.

### 2.3. Sample Efficiency: Auxiliary Loss and SCTA Components

**Figure 1:**
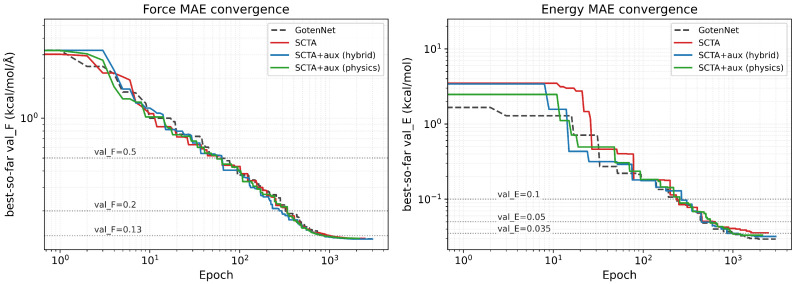
Best-so-far validation MAE vs. training epoch on rMD17 aspirin (log–log axes). Horizontal dotted lines mark the thresholds from Table 3.

**Figure 2:**
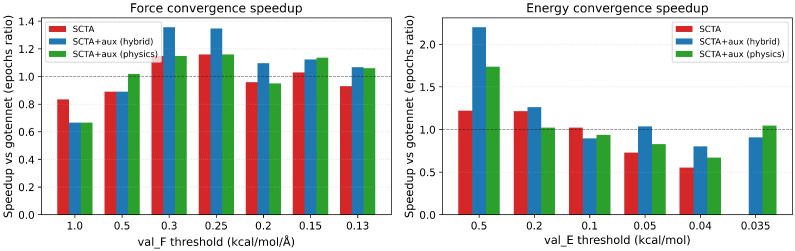
Convergence speedup relative to vanilla GotenNet at each accuracy threshold (the epoch-to-threshold ratio defined in Section 3.6). Values above the dashed 1× line indicate faster convergence. (**Left**): Force MAE thresholds. (**Right**): Energy MAE thresholds.

Because the converged test errors above differ only slightly in force MAE, we next examine whether the scalar geometric mechanisms change how quickly a usable model is obtained. We logged validation MAE every epoch and report the earliest epoch at which each model reaches a specified validation threshold (“epoch-to-threshold”) on the 50-configuration rMD17 aspirin validation split. This analysis measures training dynamics rather than final test ranking; per-epoch test evaluation is avoided at the paper-aligned scale. Results are summarized in Table 3 and visualized in Figure 1 and Figure 2.

Relative to vanilla GotenNet, SCTA alone provides a modest and threshold-dependent force acceleration, reaching val_F ∈{0.30,0.25} about 15–16% earlier but showing no consistent benefit at the earliest or tightest force thresholds. SCTA + aux provides the larger headline reductions, reaching val_F =0.25 in 211 epochs instead of 284 and val_E =0.50 in 15 epochs instead of 33. These numbers show that scalar geometric priors can shorten the path to usable validation accuracy, but do not by themselves identify which component is responsible.

To separate the SCTA branch from auxiliary supervision, Table 4 compares SCTA + aux (hybrid) with the strongest auxiliary-only baseline, GotenNet + aux (physics). This is the stricter comparison, as Section 2.1 showed that GotenNet + aux (physics) is already the best force-MAE configuration in our ablation.

Against this stronger baseline, SCTA + aux does not show a robust force-speed advantage, with the ratios moving above and below 1× depending on the threshold. The clearest speed benefit appears only at loose energy thresholds, where SCTA + aux reaches val_E ≤0.5 and ≤0.2 kcal/mol 1.5–1.8× faster than GotenNet + aux (physics), before the advantage disappears at the tighter val_E ≤0.1 threshold. Thus, the bulk of the sample efficiency gain relative to vanilla GotenNet should be attributed to the auxiliary geometric loss, while SCTA contributes a comparable scalar triplet pathway with some early-energy acceleration and a small regression at the tightest energy threshold (≤0.10 kcal/mol), rather than an advantage at single-seed precision. Sensitivity studies on angular-basis size, auxiliary-loss weight, and annealing schedule are reported in Appendix B.

### 2.4. Test Set Ablation: Base Configuration, Auxiliary Loss, and Target Type

The threshold analysis above measures training speed, but does not fully separate three design choices: the base configuration (vanilla GotenNet vs. GotenNet with SCTA), the presence of auxiliary supervision, and the auxiliary target type. Therefore, we train six configurations under the same paper-aligned protocol and evaluate each at its best-validation checkpoint. In order to test energy and force MAE, Table 5 additionally reports the validation-to-test inflation of energy MAE, (test_E−val_E)/val_E, where test_E is the test energy MAE at the best-validation checkpoint and val_E is the best (minimum) validation energy MAE reached during training. This helps to identify target choices that appear favorable on the validation split but transfer poorly to the held-out test set.

The force column reproduces the pattern observed in Section 2.1. Auxiliary supervision improves force MAE for both base configurations, and the lowest value is obtained by GotenNet + aux (physics) at 0.1280 kcal/mol/Å. Adding the SCTA capacity branch at a fixed auxiliary target changes force MAE by less than 1% and with inconsistent sign: SCTA is slightly better under the hybrid target, but slightly worse under the physics target. Therefore, the added three-body representational capacity of SCTA is not the source of the best converged force accuracy on this benchmark, which is instead provided by the auxiliary supervision.

The energy column is more informative: GotenNet + aux (physics) preserves the baseline energy accuracy (0.0357 vs. 0.0353 kcal/mol), whereas SCTA + aux (hybrid) increases test energy MAE to 0.0538 kcal/mol and shows the largest validation-to-test inflation (+69%). Switching SCTA from the hybrid target to the physics target reduces the energy inflation to +20% and the test energy MAE to 0.0397 kcal/mol, with almost no force penalty (0.1290 vs. 0.1287 kcal/mol/Å). Therefore, the hybrid target is the risky choice for SCTA, as it preserves force accuracy but overfits the small validation split on the energy axis. We attribute this to the signed dihedral mean $\bar{\cos\tau_{G}}$ in the hybrid target. As quantified in Appendix B.6 (Table A7), this component is roughly an order of magnitude smaller in terms of mean magnitude than the other two entries of the hybrid target vector (≈−0.04 vs. ≈−0.33 and ≈0.87), since the signed cosines of the many bonded dihedrals largely cancel in the graph mean. While it is not a constant or noise-like target (it retains a conformation-to-conformation CV of ≈45%), its small scale leaves it poorly matched to the other components under the shared mean-squared auxiliary loss. The SCTA branch, which mixes scalar features into the same readout pathway through its zero-initialized residual, appears to amplify this scale mismatch into the energy prediction. The physics target replaces $\bar{\cos\tau_{G}}$ with the chirality-insensitive magnitude $\bar{|\cos\tau_{G}|}$ (≈0.83), restoring a comparable scale; this is consistent with the empirical observation that switching the target stabilizes the energy axis at little force cost. A plausible direct alternative in the form of annealing the hybrid auxiliary weight to zero late in training is examined as a sensitivity probe in Appendix B rather than as a separate component.

In summary, GotenNet + aux (physics) is the strongest configuration in this ablation for peak force accuracy at minimal energy cost. SCTA remains useful as a scalar triplet design that reaches comparable force accuracy; however, when combined with auxiliary supervision, the physics target is the safer operating point.

### 2.5. Limited Cross-Molecule Probes on rMD17 Ethanol, Uracil, and Salicylic Acid

The aspirin ablation above identifies a useful operating point for this molecule, but does not provide sufficient evidence of molecule-independent improvement. To delimit the scope of the claim, we ran limited rMD17 ethanol, uracil, and salicylic acid probes and compared them with the corresponding values reported for GotenNet [46]. For salicylic acid, we additionally trained an in-house vanilla GotenNet under the same paper-aligned protocol and single seed to provide a second paired controlled comparison (alongside aspirin) at single-seed precision; ethanol and uracil are reported only against the GotenNet five-split averages. Where in-house and reported rows are compared (ethanol, uracil), the comparison is indicative rather than a fully paired multi-seed reproduction. Because the controlled aspirin ablation (Section 2.4) already shows that the SCTA capacity branch does not change converged accuracy at a fixed auxiliary setting, the cross-molecule probes focus on the auxiliary-supervised configuration; SCTA+aux is included only for ethanol as a spot check, while the uracil and salicylic acid rows report the auxiliary-only configuration (Table 6).

The cross-molecule outcome should be read as scope-limiting evidence, not as a transfer claim; we have one paired in-house comparison (salicylic acid) and two unpaired probes (ethanol, uracil).

On **salicylic acid**, we have both a paired in-house vanilla GotenNet run and the in-house GotenNet + aux (physics) run under the identical paper-aligned protocol. Auxiliary supervision *does not* reduce test error on this molecule: force MAE moves 0.06885→0.07013 kcal/mol/Å (+1.86%, with 95% analytic test set intervals [0.06765,0.07005] and [0.06898,0.07128] overlapping by about 80% of each interval half-width, so that the difference is not distinguishable from zero at finite-sample test precision), and energy MAE moves 0.01150→0.01356 kcal/mol (+17.9%, with intervals [0.00998,0.01302] and [0.01206,0.01506] in partial overlap). Therefore, the direction of the effect on salicylic acid is opposite to the direction observed on aspirin, although both differences sit close to the resolution of single-seed test-MAE comparisons. While these analytic intervals overlap, the configuration-level paired-block bootstrap of Section 2.1 resamples the correlated test configurations rather than treating force components as independent, thereby resolving this force-MAE degradation as significant ($\Delta_{F}\approx -0.00128$ kcal/mol/Å, p<0.001). The per-configuration paired differences are consistent in sign even though the marginal intervals overlap. This significance is established on a single seed, and has not been replicated across seeds.

On **uracil** and **ethanol**, no in-house paired vanilla baseline is available, so we compare in-house GotenNet + aux (physics) only against the reported GotenNet five-split averages: uracil energy MAE moves 0.0064→0.00577 kcal/mol (−9.8%) and force MAE 0.0417→0.04248 kcal/mol/Å (+1.9%); ethanol 0.0071→0.00741 kcal/mol (+4.4%) and 0.0482→0.05022 kcal/mol/Å (+4.2%). These unpaired directional differences are within the range expected when a single-split and single-seed in-house run is compared against five-split averages; thus, we report the magnitudes but do not treat the signs as evidence of transfer. SCTA + aux (physics) on ethanol provides 0.05186 vs. 0.05022 for the auxiliary-only run, consistent with the aspirin finding that the SCTA capacity branch does not add converged accuracy.

Across the two paired controlled comparisons (aspirin, salicylic acid) and the two unpaired probes (ethanol, uracil), the auxiliary effect lacks a uniform direction. Under the same configuration-level paired block bootstrap, the aspirin single-seed force improvement is not by itself distinguishable from test set sampling noise; instead, its robust support comes from the three-seed variance reduction of Section 2.2), whereas the salicylic degradation is significant (p<0.001, though likewise on a single seed). Therefore, the directional inconsistency between the two molecules is statistically resolved rather than an artifact of the test set resolution. We treat this directional inconsistency as the cross-molecule finding, and it constrains the conclusion of this paper; that is, the auxiliary loss is the more effective lever on aspirin under the protocol tested, with mixed or null evidence on the other rMD17 molecules examined here. The most likely explanation for the small magnitudes on the unpaired probes is the absolute error scale. The converged force MAE on ethanol (≈0.05 kcal/mol/Å) is already roughly 2.6× lower than on aspirin (≈0.13 kcal/mol/Å), and the strongest models in the GotenNet benchmark (NequIP, MACE, and Allegro) all cluster within roughly 0.048–0.065 kcal/mol/Å on ethanol force [46], leaving little headroom for any low-cost modification. A degenerate supervision signal is not the explanation here; the conformation-to-conformation variance of the auxiliary geometric target on ethanol is comparable to or larger than on aspirin (Appendix B.6), so the target itself carries usable information.

### 2.6. Emergent Layer-Wise Self-Gating

**Figure 3:**
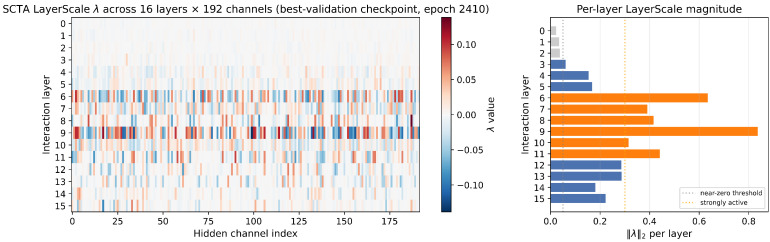
Trained SCTA LayerScale $\lambda^{\left(l\right)}$ across 16 interaction layers and 192 hidden channels on rMD17 aspirin at the best-validation checkpoint (epoch 2410). (**Left**): Per-channel heatmap. (**Right**): Per-layer $∥\lambda^{\left(l\right)}{∥}_{2}$. Bar color encodes activation level (orange: strongly active; blue: intermediate; gray: near-zero); dotted lines mark the two thresholds.

SCTA is attached through a zero-initialized LayerScale residual, $h^{\left(l+1\right)}\leftarrow h^{\left(l+1\right)}+\lambda^{\left(l\right)}⊙{\mathrm{SCTA}}^{\left(l\right)}\left(\cdot \right)$, so the trained values of $\lambda^{\left(l\right)}$ indicate where the optimizer chooses to engage the triplet branch. Evaluated at the best-validation checkpoint, the learned pattern is strongly depth-dependent (Figure 3): the earliest layers L0–L2 remain almost off ($∥\lambda^{\left(l\right)}{∥}_{2}<0.04$), the branch is strongly active across the middle block L6–L11 ($∥\lambda^{\left(l\right)}{∥}_{2}\in \left[0.32,0.84\right]$), and the final layers L12–L15 decay back to a moderate level ($∥\lambda^{\left(l\right)}{∥}_{2}\in \left[0.18,0.29\right]$) without returning to zero. Therefore, SCTA behaves as a mid-depth geometric correction rather than as a uniformly active replacement for the backbone’s implicit angular pathway. This depth-dependent pattern is specific to the 16-layer paper-aligned configuration: in the pilot configurations with 2–3 layers used in Appendix A and Appendix B.1, all SCTA layers necessarily occupy similar relative depths, meaning that the mid-depth interpretation does not apply.

### 2.7. Pilot Ablation: SCTA Does Not Replace Tensor Features

The self-gating pattern raises a narrower architectural question: can the explicit scalar triplet branch compensate for reducing the backbone’s steerable-feature order? To test this, we ran a reduced pilot ablation on aspirin ($d_{ne}=32$, 3 interaction layers, 100 epochs, single seed 42), comparing GotenNet with $L_{\max}=2$ or $L_{\max}=1$ and with or without SCTA. This pilot is intended as a qualitative architecture check, not as a replacement for the paper-aligned 3000-epoch results.

The pilot result is clear in its direction: reducing the backbone from $L_{\max}=2$ to $L_{\max}=1$ worsens force MAE by 42% (A → B). Adding SCTA to this weakened backbone does not recover the lost tensor channel; it further degrades force MAE in this short-budget setting (B → C). In contrast, adding SCTA to the full $L_{\max}=2$ backbone improves the pilot validation errors (A → D). The conclusion is not that SCTA can simplify the backbone, but that it is useful only when the underlying equivariant representation remains strong enough. At this 100-epoch pilot budget, val_E does not converge and is sensitive to architectural perturbations (compare Appendix B.5). Thus, the force column should be read as the more stable indicator at pilot scale (Table 7).

This result matches the design intent of SCTA. The branch supplies an explicit scalar cosine-angle signal, whereas the l=2 steerable channel carries higher-order angular information that is not reducible to a single triplet cosine. Therefore, SCTA should be treated as a residual scalar three-body prior on top of a competent equivariant backbone rather than as a substitute for the backbone’s tensor features.

### 2.8. Note on Frame-Projection Alternatives

A natural control for SCTA is to replace its explicit $\cos\theta_{jik}$ input with scalars obtained by projecting equivariant tensor features onto a local triplet frame, following the spirit of LEFTNet [50], frame averaging [51], and related inertial-frame designs [52]. At pilot configuration C, the frame projection branch is empirically competitive with SCTA. In parallel with SCTA, it gives essentially the same force MAE (1.85 vs. 1.85 kcal/mol/Å); as a full replacement for SCTA, it matches SCTA on force (1.83) while noticeably reducing energy MAE (0.87 vs. 1.24 kcal/mol; see full table in Appendix A). Therefore, we do not present frame projection as a failure case at this scale.

The algebraic analysis adds a design rationale rather than an explanation of an empirical failure. Under any orthonormal triplet frame, the diagonal projected pairwise scalar collapses to the ordinary tensor inner product $\langle X_{j},X_{k}\rangle$, so it does not encode the chosen local frame. The remaining genuinely frame-dependent channel is a pseudoscalar triple product, so it is not an O(3) scalar of the kind that a parity-even energy target on achiral molecules can directly exploit. The cos-angle input of SCTA avoids both issues by construction, being a true O(3)-invariant three-body scalar that requires no frame, which is why we adopt it as the main design in this paper. The full setup, empirical table, and proofs are given in Appendix A.

### 2.9. Computational Cost

#### 2.9.1. Per-Layer Asymptotic Cost

Because scalability is a central constraint for neural interatomic potentials [53], we report both asymptotic and measured costs. SCTA adds two operations to each forward pass. First, triplet enumeration is performed once and cached across interaction layers, requiring |T|=∑i=1Ndi2 triplet index tuples for node degrees $d_{i}$. Second, each layer applies triplet attention: element-wise feature products, softmax over triplets grouped by center, scatter aggregation, and a final linear projection. This layer-wise branch has complexity O(|T|·D) in hidden dimension *D*. No Clebsch–Gordan coupling appears, so the classical tensor-product scaling factor $O(L^{6})$ is absent.

By comparison, each Graph Attention Transformer Architecture (GATA) layer in the GotenNet backbone has a cost of O(|E|·D) on the edges $\left|E\right|=\sum_{i}d_{i}$. Therefore, the SCTA-to-GATA asymptotic ratio is

(1) SCTAper-layercostGATAper-layercost≈∑idi2∑idi≈d¯−12,

where $\bar{d}=\langle d_{i}\rangle$ is the average node degree. Equation (1) is obtained in two steps that make the two “≈” explicit. The first step cancels the common factor *D* in the two per-layer costs, O(|T|·D)/O(|E|·D)=|T|/|E|, and substitutes the exact counts |T|=∑idi2 and $\left|E\right|=\sum_{i}d_{i}$. The second step replaces this ratio of degree-dependent sums by a single function of the mean degree: writing di2=12di(di−1) gives ∑idi2/∑idi=12〈di2〉/d¯−1, which reduces to $(\bar{d}-1)/2$ when the degree distribution is concentrated around $\bar{d}$ (i.e., $\langle d_{i}^{2}\rangle \approx {\bar{d}}^{2}$). The residual term $\frac{1}{2}\left(\langle d_{i}^{2}\rangle -{\bar{d}}^{2}\right)/\bar{d}$ is a non-negative degree variance correction, so $(\bar{d}-1)/2$ is a lower bound on the true triplet-to-edge ratio. On rMD17 aspirin (21 atoms, 5 Å radial cutoff), $\bar{d}\approx 14$, for a nominal triplet-to-edge ratio of ≈6.5×. Numerically, each aspirin graph has |T|≈1911 triplets (≈91 per atom) and $\left|E\right|=\sum_{i}d_{i}\approx 294$ directed edges, so the exact ratio |T|/|E|≈6.5 coincides with the approximation $(\bar{d}-1)/2\approx 6.5$ to the quoted precision, confirming that correction of the degree variance is negligible for this near-homogeneous neighborhood. The measured epoch-level overhead is much smaller because GATA also contains steerable-feature updates and heavier pairwise message MLPs, whereas SCTA uses a lightweight scalar attention branch. Therefore, the asymptotic ratio in Equation (1) upper-bounds rather than predicts the realized per-epoch cost measured next.

#### 2.9.2. Empirical Wall Clock

At the paper-aligned configuration ($d_{ne}=192$, 16 layers) on a single NVIDIA RTX 6000 Ada with batch size 4, one pass through the 950-sample aspirin training split takes approximately 48 s/epoch for vanilla GotenNet and 62 s/epoch for SCTA (single timing run, not repeated), corresponding to a ∼29% per-epoch overhead from the triplet branch. This measured 29% is far below the ≈6.5× asymptotic triplet-to-edge ratio of Equation (1). This is because the per-epoch wall clock is dominated by the backbone’s steerable-feature (HTR) updates and pairwise message MLPs, against which the added scalar triplet work O(|T|·D) is comparatively cheap; the asymptotic ratio counts triplets relative to edges, not relative to the backbone’s full per-edge tensor workload. The cross-molecule probes of Section 2.5 were trained on a separate single NVIDIA RTX 4090; the hardware choice affects only wall clock timing, not the reported MAE values. Relative to vanilla GotenNet, SCTA + aux can still reduce wall clock time at selected early-to-mid validation thresholds, since it needs fewer epochs: val_F =0.25 kcal/mol/Å is reached at approximately 211×62s≈3.6 GPU-hours for SCTA + aux vs. 284×48s≈3.8 GPU-hours for vanilla GotenNet, and val_E =0.5 kcal/mol is reached in 15×62s≈0.26 h vs. 33×48s≈0.44 h.

This comparison should not be read as a general SCTA throughput advantage. Section 2.3 shows that the vanilla comparison conflates SCTA and auxiliary supervision and that SCTA + aux does not have a robust force time-to-threshold advantage over the stronger GotenNet + aux (physics) baseline. Therefore, the compute-side conclusion is narrower: auxiliary geometric supervision provides most of the sample efficiency benefit at negligible cost, while SCTA introduces moderate triplet overhead and is justified mainly as a no-CG scalar three-body design and as an analytical probe.

#### 2.9.3. Auxiliary-Loss Overhead

The auxiliary geometric loss adds negligible overhead. The graph-level target is computed under torch.no_grad once per batch, and the two-layer auxiliary-head Multi-Layer Perceptron (MLP) adds ≪1% of GotenNet’s total parameter count. Therefore, the auxiliary loss is practically “free” at inference time (the auxiliary head is not used for energy/force prediction) and contributes only an additional <0.5 s per epoch during training.

#### 2.9.4. Memory

The dominant memory cost of the SCTA branch is the triplet-index cache (precomputed once per forward pass and reused across all interaction layers), which scales as O(|T|) with the same triplet-to-edge ratio $(\bar{d}-1)/2$ as the FLOP cost. At rMD17 aspirin ($\bar{d}\approx 14$, |T|≈1900 per graph) and batch size 4, the cached triplet tensors occupy a small fraction of activation memory relative to the steerable feature buffers used by the Hierarchical Tensor Refinement (HTR) module; we did not observe a memory-bound regime for this benchmark, although for larger systems with denser neighborhoods |T| would scale quadratically with $\bar{d}$ and could become limiting. Concretely, the cache holds only a few integer index tensors per triplet (the center, its two neighbors, and the two contributing edges) shared across all 16 interaction layers, so its footprint is negligible other than the floating-point activation buffers. For large or periodic systems in which |T| grows quadratically with $\bar{d}$, capping the neighbor count or enumerating triplets in blocks would bound this cost without changing the scalar attention itself.

## 3. Materials and Methods

**Figure 4:**
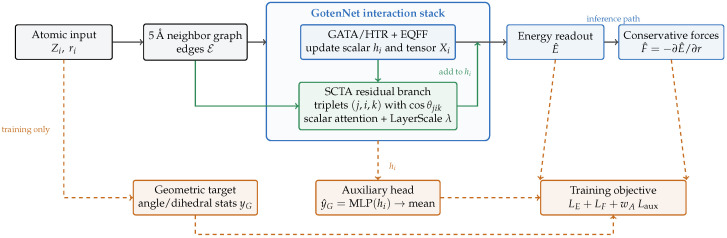
Architecture overview of the proposed GotenNet extension, separating the inference-time energy–force path (top, blue) from the training-only auxiliary-supervision path (bottom, orange, dashed). The SCTA residual branch (green) is inserted inside the interaction stack.

Figure 4 provides an overview of the implemented architecture and separates the inference-time energy–force path from the training-only auxiliary supervision path. Along the inference path (top row of Figure 4), atomic inputs $\left(Z_{i},r_{i}\right)$ build a 5 Å neighbor graph that feeds the GotenNet interaction stack; the stack’s scalar features $h_{i}$ are read out as the energy $\hat{E}$ and differentiated to give conservative forces $\hat{F}=-\partial \hat{E}/\partial r$. The SCTA residual branch sits inside this stack, adding a scalar triplet-attention term to $h_{i}$ (Section 3.3). The bottom row is active only during training: a geometric target on bond-angle and dihedral statistics (Section 3.4), an auxiliary head that reads $h_{i}$, and the combined objective $L_{E}+L_{F}+w_{A}L_{\mathrm{aux}}$ (Section 3.5). The auxiliary head is discarded at inference, so it shapes the representation during training without adding any test-time cost.

### 3.1. Dataset and Units

We use the **rMD17** dataset [47], a revised version of MD17 [5] with DFT energies and forces for small organic molecules at the Perdew–Burke–Ernzerhof (PBE)/def2-SVP level. The main controlled experiments use aspirin ($C_{9}H_{8}O_{4}$, 21 atoms, 100,000 configurations). Following the GotenNet evaluation protocol [46], we use the 950/50/1000 train/validation/test split and keep the corresponding split file (splits_0.npz) fixed for all in-house comparisons. Section 2.5 adds limited ethanol, uracil, and salicylic acid probes using the same code path and compares them with the corresponding GotenNet reported values; these probes are used only to bound the transfer claim.

During training and evaluation, energies and forces are handled in the dataset’s native kcal/mol and kcal mol−1 Å−1 units, matching the convention used in GotenNet. Literature baselines are converted to these units when necessary. Energies are standardized to zero mean and unit variance using the training set; forces are not standardized.

### 3.2. Backbone: GotenNet

We use GotenNet [46] as the equivariant backbone. Given atomic numbers $z\in N^{N}$ and positions $r\in R^{N\times 3}$, the model constructs a 5 Å radial-cutoff graph and maintains scalar node features $h_{i}\in R^{D}$ together with steerable tensor features $X_{i}\in R^{({\left(\ell_{\max}+1\right)}^{2}-1)\times D}$. Unless otherwise stated, the paper-aligned runs use D=192, 16 interaction blocks, and $\ell_{\max}=2$.

Each interaction block applies GATA with HTR edge updates followed by Equivariant Feed-Forward (EQFF) updates to the scalar and steerable channels. SCTA and the auxiliary head are added around this backbone without changing the backbone energy readout. The final scalar node features are mapped to atomic energy contributions by a two-layer MLP and summed over atoms, giving the standardized energy readout

(2) $$\tilde{E}=\sum_{i=1}^{N}s_{\theta }\left(h_{i}\right),\;$$

where $s_{\theta }$ denotes the two-layer atom-wise MLP acting on the final scalar features $h_{i}$ and $\tilde{E}$ is the energy in the standardized units of Section 3.1. Forces are computed as F=−∂E/∂r, preserving energy–force consistency.

### 3.3. Scalarization-Compatible Triplet Cross-Attention (SCTA)

The backbone of Section 3.2 is held fixed throughout. The two mechanisms examined in this work are introduced around it. The first (this subsection) augments the model with explicit three-body representational capacity through a scalar triplet branch (SCTA); the second (Section 3.4) instead augments the training supervision through an auxiliary geometric loss. Because both are attached to an identical backbone, the capacity-versus-supervision comparison of Section 2 is a controlled one.

Each SCTA layer chains five steps. First, the triplet angle is embedded in a fixed angular basis (Equation (3)). This basis gates a per-triplet attention score (Equation (4)), which is then normalized over the triplets sharing a center (Equation (5)). The resulting weights aggregate a symmetric neighbor value (Equation (6)), and the aggregated message is added back to the scalar channel through a zero-initialized LayerScale residual (Equation (7)).

SCTA is a scalar residual branch inserted after GATA and EQFF in each interaction block. For each center atom *i*, we enumerate unordered neighbor pairs (j,k) and form triplets (i,j,k) with j≠k. The branch uses only scalar node features and the angle cosine $\cos\theta_{jik}={\hat{r}}_{ij}\cdot {\hat{r}}_{ik}$; it does not introduce Clebsch–Gordan tensor products or alter the steerable tensor channel directly.

#### 3.3.1. Triplet Enumeration

Triplet indices are built once per forward pass and cached across all interaction blocks. For a node with graph degree $d_{i}$, the branch enumerates di2 neighbor pairs, giving |T|=∑idi2 triplets in total. On rMD17 aspirin at the 5 Å cutoff, this corresponds to about 91 triplets per atom on average.

#### 3.3.2. Angular Basis

The angle is encoded by a fixed Gaussian basis on cosθ∈[−1,1]:

(3) $$\phi_{n}\left(\cos\theta \right)=\exp\;\left(-\gamma \;{\left(\cos\theta -\mu_{n}\right)}^{2}\right),\;n=1,\ldots ,N_{\mathrm{ang}}\;$$

where $\mu_{n}$ are uniformly spaced and $\gamma =2{\left(N_{\mathrm{ang}}-1\right)}^{2}$. We use $N_{\mathrm{ang}}=8$ throughout. The angular basis is projected to hidden dimension *D* by a bias-free linear map $\psi \left(\theta \right)=W_{\phi }\phi \left(\cos\theta \right)$, where $\psi_{c}$ denotes the *c*-th channel of ψ. Equation (3) is not evaluated globally or pooled over the graph, instead being computed independently for each triplet (j,i,k) from that triplet’s own angle cosine $\cos\theta_{jik}={\hat{r}}_{ij}\cdot {\hat{r}}_{ik}$. The resulting per-triplet vector $\psi (\theta_{jik})$ is exactly the quantity that enters the attention score in Equation (4) below. Therefore, the angular basis acts only as a local geometric gate on each triplet, not as a graph-level descriptor.

#### 3.3.3. Attention

Following scaled dot-product attention [54], we compute query, key, and value projections from scalar node features: $q_{i}=W_{q}h_{i}$, $k_{j}=W_{k}h_{j}$, and $v_{j}=W_{v}h_{j}$, where $W_{q},W_{k},W_{v}\in R^{D\times D}$ are learned weight matrices acting on the scalar node features *h*. The scalar score for triplet (i,j,k) is

(4) $$s_{ijk}=\frac{1}{\sqrt{D}}\sum_{c=1}^{D}q_{i,c}\;k_{j,c}\;k_{k,c}\;\psi_{c}\left(\theta_{jik}\right),\;$$

where *c* indexes the *D* hidden channels, $q_{i,c}$, $k_{j,c}$, $k_{k,c}$ are the *c*-th components of the center query and the two neighbor keys, $\psi_{c}\left(\theta_{jik}\right)$ is the *c*-th channel of the projected angular basis of Equation (3), and $1/\sqrt{D}$ is the standard scaled-dot-product normalization that keeps the variance of $s_{ijk}$ approximately constant in *D*. The four-way product $q_{i,c}\;k_{j,c}\;k_{k,c}\;\psi_{c}$ is the scalar j↔k-symmetric analogue of a pairwise dot-product attention logit, and is large only when the center, both neighbors, and the triplet angle are mutually aligned in channel *c*. The score is followed by a softmax over all triplets centered at the same atom:

(5) $$w_{ijk}={\mathrm{softmax}}_{\left(j,k\right)\in T_{i}}\left(s_{ijk}\right)\;=\;\frac{\exp(s_{ijk})}{\sum_{\left(j^{\prime },k^{\prime }\right)\in T_{i}}\exp\left(s_{ij^{\prime }k^{\prime }}\right)}$$

where $T_{i}=\left\{\left(j,k\right):j,k\in N\left(i\right),\;j\neq k\right\}$ is the set of neighbor pairs around the center *i*, so that $\sum_{\left(j,k\right)\in T_{i}}w_{ijk}=1$ and the branch forms a convex per-center combination of triplet messages.

#### 3.3.4. Aggregation

The triplet message is aggregated symmetrically over (j,k):

(6) $${\tilde{h}}_{i}=\sum_{\left(j,k\right)\in T_{i}}w_{ijk}\;\left(v_{j}+v_{k}\right)\;$$

and passed through an output projection before being added to the scalar channel:

(7) $$h_{i}\leftarrow h_{i}+\lambda ⊙W_{\mathrm{out}}\;{\tilde{h}}_{i}.\;$$

The LayerScale vector $\lambda \in R^{D}$ is initialized to zero so the SCTA branch is exactly inactive at initialization; therefore, its learned magnitude reveals where the optimizer chooses to engage the explicit triplet signal.

### 3.4. Auxiliary Geometric Supervision

Whereas SCTA enlarges the inference-time representational capacity of the model (Section 3.3), the auxiliary supervision introduced here leaves the inference network unchanged and instead shapes the learned representation through an additional training objective. The auxiliary path follows a chain parallel to that of SCTA: a bonded subgraph is identified (Equation (8)), reduced to a low-dimensional geometric target in one of two variants (Equations (9) and (10)), regressed by a graph-level auxiliary head (Equation (11)), and trained against that target using a mean-squared error (Equation (12)).

The auxiliary head applies graph-level supervision to scalar representations using geometric summaries computed from the molecular structure. It is used only during training, and is removed from the energy/force prediction path at inference time. The motivation for the construction below is that the quantities supervised by the auxiliary loss (bond angles and dihedrals) are defined over *covalent bonds*, whereas the 5 Å radial-cutoff graph of Section 3.2 also connects many non-bonded atom pairs. Enumerating angles and torsions on that message-passing graph would mix chemically meaningful bond angles with spurious through-space angles, which we avoid by evaluating the geometric targets on a separate bonded subgraph rather than on the message-passing graph. We first identify a bonded subgraph

(8) $$B_{G}=\{\left(i,j\right):∥r_{i}-r_{j}∥<1.8\;Å,\;i\neq j\}\;$$

which is separate from the 5 Å message-passing graph. The 1.8 Å threshold is a conservative upper bound for typical single covalent bonds in the rMD17 molecules considered here (e.g., C–H ≈1.10 Å, C–C ≈1.54 Å, C–O ≈1.43 Å, N–H ≈1.01 Å, O–H ≈0.96 Å). It sits above the longest of these single bonds yet below the shortest non-bonded contact distance, so that the bonded subgraph approximates the chemical bond graph without explicit bond perception, valence assignment, or a chemistry toolkit. The cutoff is used as a fixed hyperparameter rather than being tuned per molecule. Moreover, the threshold is supported empirically: the explicit ablation in Appendix B.2 (Table A3) shows that 1.8 Å is the best of the three tested values {1.6,1.8,2.0} Å on *both* the energy and force axes at pilot configuration C, with a force-MAE spread of only ∼1% across the sweep, so the threshold is not finely tuned. The bonded triplets $\theta_{G}$ and quadruplets $\tau_{G}$ entering Equations (9) and (10) are enumerated on $B_{G}$, not on the message-passing graph.

We evaluate two three-dimensional target variants. The *hybrid* target summarizes signed bond-angle and dihedral statistics

(9) $$y_{G}^{\mathrm{hyb}}={\left[\;\bar{\cos\theta_{G}},\;\bar{\cos\tau_{G}},\;\mathrm{std}\left(\cos\tau_{G}\right)\;\right]}^{⊤},\;$$

where $\theta_{G}$ ranges over bonded triplets and $\tau_{G}$ over bonded quadruplets. For a quadruplet (i,j,k,l), $\cos\tau =\left(n_{1}\cdot n_{2}\right)/(∥n_{1}∥∥n_{2}∥)$ with $n_{1}=r_{ij}\times r_{jk}$ and $n_{2}=r_{jk}\times r_{kl}$. One component of the hybrid target is the signed dihedral mean $\bar{\cos\tau_{G}}$, which is poorly scaled; it is small in magnitude (about −0.04 on aspirin, roughly an order of magnitude below the other two components), which is because the signed cosines of the many bonded dihedrals largely cancel in the graph mean (Appendix B.6). This scale mismatch is the most likely source of the energy-axis instability seen when the hybrid target is combined with SCTA (Section 2.4). We did not directly isolate the scale from sensitivity to chirality, for example by rescaling $\bar{\cos\tau_{G}}$ alone, so the attribution rests on the component statistics together with the stabilizing effect of replacing the signed mean by its magnitude in the physics target. The *physics* target instead replaces the signed dihedral mean with chirality-insensitive torsion magnitude and bond-length summaries

(10) $$y_{G}^{\mathrm{phys}}={\left[\;\bar{|\cos\tau_{G}|},\;d_{\min,G},\;s_{\mathrm{bond},G}\;\right]}^{⊤},\;$$

where $d_{\min,G}$ is the minimum bonded distance and $s_{\mathrm{bond},G}$ is the mean over atoms of the standard deviation of incident bonded distances.

Both target variants are computed under torch.no_grad; thus, the auxiliary loss supervises the representation but does not add a direct force-gradient term through the geometric target.

A two-layer MLP maps the per-node scalar features to the auxiliary dimension, followed by a graph-level mean pooling

(11) $${\hat{y}}_{G}={\mathrm{mean}}_{i\in G}\;\mathrm{MLP}\left(h_{i}\right).\;$$

We use a graph-mean pooling head matched to a graph-level target $y_{G}$ rather than per-bond or per-triplet local supervision (e.g., histogram-matching on bond-angle distributions or edge-level prediction of $\cos\theta_{jik}$) for two practical reasons: it aligns with the graph-level mean readout already used by the GotenNet backbone for energy, and it does not commit the model to a specific bond/triplet partition during representation learning. This graph-level design is coarse by choice, as it supervises only aggregate geometric statistics, not *where* a geometric error occurs. In Appendix B.7, we discuss more localized finer-grained alternatives per-bond or per-triplet targets, distribution matching, and per-atom descriptors) along with their drawbacks and why we leave them to future work. The auxiliary loss is the mean-squared error, with $y_{G}$ denoting either target variant:

(12) $$L_{\mathrm{aux}}=∥{\hat{y}}_{G}-y_{G}{∥}_{2}^{2}.$$

### 3.5. Overall Training Objective

The standardized energy prediction is mapped back to physical units and differentiated into conservative forces (Equation (14)), which enter the combined energy–force objective together with the auxiliary term of Section 3.4 (Equation (15)).

The training objective follows the GotenNet energy–force loss, and adds the auxiliary geometric term only when the corresponding head is enabled. Let $\tilde{E}$ denote the standardized energy prediction of Equation (2) and ${\tilde{E}}^{*}$ the standardized DFT energy target, where standardization uses the training set energy mean $\mu_{E}$ and standard deviation $\sigma_{E}$ of Section 3.1 through ${\tilde{E}}^{*}=\left(E^{*}-\mu_{E}\right)/\sigma_{E}$. The physical energy is recovered by inverting this standardization:

(13) $$\hat{E}=\sigma_{E}\;\tilde{E}+\mu_{E}$$

with $\mu_{E},\sigma_{E}$ as the same training set statistics. Forces then follow from this physical energy by energy conservation. Because $\mu_{E}$ and $\sigma_{E}$ are constants independent of *r*, the additive shift $\mu_{E}$ vanishes under the derivative and the scale $\sigma_{E}$ factors out:

(14) $$\hat{F}=-\frac{\partial \hat{E}}{\partial r}\;=\;-\sigma_{E}\;\frac{\partial \tilde{E}}{\partial r}\;$$

so that energy and force predictions share a single scalar potential and the force scale is fixed by $\sigma_{E}$ rather than being learned independently. The full loss is

(15) $$L=w_{E}\;∥\tilde{E}-{\tilde{E}}^{*}{∥}^{2}+w_{F}\;{∥\hat{F}-F^{*}∥}^{2}+w_{A}\left(t\right)\;L_{\mathrm{aux}},\;$$

where $F^{*}$ is the reference DFT force in kcal/mol/Å. We use $w_{E}=0.05$ and $w_{F}=0.95$ for all in-house runs. For non-auxiliary models, we set $w_{A}\left(t\right)=0$. Auxiliary-supervised models use the constant setting $w_{A}\left(t\right)=0.02$ in the main paper-aligned experiments; the sensitivity study also tests a linear decay during the first $T_{\mathrm{anneal}}$ epochs:

(16) $$w_{A}\left(t\right)=0.02\cdot \max\;\left(0,\;1-t/T_{\mathrm{anneal}}\right).\;$$

The auxiliary head is discarded for energy and force evaluation; thus, the auxiliary target shapes the learned representation during training but does not introduce a separate inference-time prediction path.

### 3.6. Training Protocol

All paper-aligned models are trained with AdamW [55,56] at learning rate $2\times 10^{-4}$, with 1000 warmup steps and a ReduceLROnPlateau schedule to monitor the validation loss (patience 30 epochs, decay factor 0.8, minimum learning rate $10^{-7}$). Weight decay is set to zero. Exponential Moving Averaging (EMA) of model weights with decay rate 0.9 is used to compute the validation metrics, and is the set of weights restored at the best-validation checkpoint for the test evaluation reported in Table 1 and Table 5; this choice is fixed across all six in-house configurations, and does not bias the auxiliary-versus-no-auxiliary comparison. Each model is trained for up to 3000 epochs with batch size 4 and inference batch size 4 on a single NVIDIA RTX 6000 Ada GPU with 96 GB memory, and is evaluated at the checkpoint with the best validation loss. Actual training durations range from ∼2100 to 3000 epochs across the six configurations of Table 1, and the full per-epoch metric logs are released as part of the data package. The train/validation/test split and random seed are fixed across all in-house comparisons.

#### 3.6.1. Error Metrics

All energy and force errors reported in this paper, including the values tabulated in Table 1 and Table 5, consist of Mean Absolute Error (MAE) evaluated on the held-out test split. For a set of $N_{\mathrm{test}}=1000$ test configurations indexed by *n*, the energy MAE is

(17) $${\mathrm{MAE}}_{E}=\frac{1}{N_{\mathrm{test}}}\sum_{n=1}^{N_{\mathrm{test}}}|\;{\hat{E}}_{n}-E_{n}^{*}\;|,\;$$

where ${\hat{E}}_{n}$ is the model energy and $E_{n}^{*}$ the DFT reference energy, both in kcal/mol. The force MAE averages over every Cartesian force component of every atom in every test configuration:

(18) $${\mathrm{MAE}}_{F}=\frac{1}{3\;A\;N_{\mathrm{test}}}\sum_{n=1}^{N_{\mathrm{test}}}\sum_{a=1}^{A}\sum_{c\in \{x,y,z\}}|\;{\hat{F}}_{n,a,c}-F_{n,a,c}^{*}\;|\;$$

where *A* is the number of atoms (A=21 for aspirin), ${\hat{F}}_{n,a,c}$ is the predicted force, and $F_{n,a,c}^{*}$ is the DFT reference in kcal/mol/Å. For aspirin, this yields 3×21×1000=63,000 force components, which is the sample size *n* used for the analytic force confidence intervals in Section 2.1. The validation MAE used for checkpoint selection and for the sample efficiency analysis of Section 2.3 is defined identically but evaluated on the 50-configuration validation split.

The test MAE values in Table 1 and Table 5 are evaluated once at the checkpoint with the best validation loss. Per-epoch test metrics are not used for checkpoint selection or sample-efficiency analysis.

Reduced-capacity runs are used only for qualitative ablations and supplementary checks. Section 2.7 uses hidden dimension 32, three interaction layers, and $L_{\max}\in \left\{1,2\right\}$ to probe tensor-feature complementarity. Appendix A uses hidden dimension 16 and two interaction layers for frame-projection controls. These pilot configurations are trained for 100 epochs on CPU with seed 42, and otherwise follow the same optimizer and scheduler settings.

#### 3.6.2. Sample Efficiency Metric

To quantify convergence behavior for the auxiliary and SCTA variants (Section 2.3), we record the validation energy and force MAE at the end of every training epoch during the paper-aligned 3000-epoch runs; for each model, we compute the earliest epoch $t^{⋆}$ at which the best-so-far validation MAE reaches a given threshold τ:

(19) $$t^{⋆}\left(\tau \right)=\min\left\{\;t\in \left\{1,\ldots ,3000\right\}\;:\;\min_{s\leq t}\;{\mathrm{MAE}}_{\mathrm{val}}\left(s\right)\leq \tau \;\right\}\;$$

where ${\mathrm{MAE}}_{\mathrm{val}}\left(s\right)$ is the validation MAE recorded at epoch *s*. The inner $\min_{s\leq t}$ takes the best (lowest) value seen up to epoch *t*, while the outer minimum returns the first epoch index meeting the threshold; thus, $t^{⋆}\left(\tau \right)$ is a single integer in {1,…,3000} rather than a continuous quantity. Thresholds are expressed in the same native units as the reported MAE values (kcal/mol for energy and kcal/mol/Å for force). The speedup of model *M* relative to GotenNet is the ratio of the two integer epoch counts obtained from Equation (19):

(20) $$S_{M}\left(\tau \right)=\frac{t_{\mathrm{GotenNet}}^{⋆}\left(\tau \right)}{t_{M}^{⋆}\left(\tau \right)}.\;$$

Therefore, the right-hand side involves no integration, as $t_{\mathrm{GotenNet}}^{⋆}\left(\tau \right)$ and $t_{M}^{⋆}\left(\tau \right)$ are the respective integer epochs at which vanilla GotenNet and model *M* first cross the threshold τ, and $S_{M}\left(\tau \right)$ is simply their quotient. Values above 1 indicate faster convergence than vanilla GotenNet. If a run does not reach a threshold within 3000 epochs, no speedup is reported for that threshold.

### 3.7. Implementation

We implement SCTA and the auxiliary loss on top of the public GotenNet codebase using PyTorch 2.5.1, PyTorch Geometric 2.7.0, PyTorch Lightning 2.2.5, e3nn 0.6.0, and Hydra 1.3.2. SCTA is added as a representation module extension after each GATA/EQFF block; triplet indices are generated from the same 5 Å neighbor graph, cached once per forward pass, and reused across interaction layers. The auxiliary target construction is performed under torch.no_grad, and the auxiliary head contributes only to the training loss.

Hydra configuration files specify the backbone, hidden size, number of interaction layers, whether SCTA is enabled, auxiliary target type (hybrid or physics), auxiliary weight, and training budget. Source code, trained checkpoints, configuration files, and experimental logs will be released upon acceptance.

## 4. Conclusions

This work asks whether the difficulty of capturing three-body geometry in a no-Clebsch–Gordan backbone such as GotenNet is a matter of representational capacity or of training supervision. We address the question with three controlled probes on a single-seed paper-aligned rMD17 aspirin split: two that add representational capacity (frame projection of tensor features and scalarization-compatible triplet cross-attention) and one that adds supervision instead of capacity (a graph-level auxiliary loss on bond-angle and dihedral statistics).

Frame projection is included as a natural alternative scalar three-body branch. At pilot configuration C, it performs comparably to SCTA on aspirin: when used in parallel with SCTA, the two branches give the same force MAE; when used as a replacement for SCTA, the frame branch matches SCTA on force and noticeably reduces energy MAE. Algebraically, however, the frame projection’s two scalar outputs have specific structural limitations: the diagonal projected feature is exactly frame-independent and reduces to an ordinary tensor inner product, and the only genuinely frame-dependent channel is parity-odd. These observations motivate the cos-angle input of SCTA as the more principled scalar three-body choice, since it is a true O(3)-invariant of the triplet and requires no frame construction.

As a correctly designed scalar triplet branch that avoids both algebraic limitations of frame projection, SCTA matches the converged force accuracy of the GotenNet backbone to within ∼0.4% on aspirin; however, it does not produce a robust independent gain over the stronger GotenNet + aux (physics) baseline. We read this neutral outcome as evidence that the backbone’s implicit angular pathway already supplies the relevant three-body representational capacity, an interpretation that is supported by the learned LayerScale weights (nearly inactive in early layers, strongly active in the middle interaction blocks at paper-aligned scale). Therefore, adding more capacity is not the binding constraint, even when correctly scalarized. A pathway-level probe (Appendix B.5) supports the same picture from the opposite side: removing the l=1⊗l=1 inner-product term from GotenNet’s HTR pathway costs almost nothing on pilot val_F and noticeably improves pilot val_E, suggesting that the backbone’s existing angular capacity is if anything not monotonically beneficial across both prediction targets at small scale. We leave this observation as an opening for future architectural simplification.

Of the two levers, supervision is the one with a measurable effect on converged accuracy, though a modest and seed-dependent one. On the single-seed ablation, the physics-style auxiliary target gives the lowest force MAE (0.1280 kcal/mol/Å vs. 0.1303 for reproduced GotenNet); across three random seeds (Section 2.2), it lowers the mean force MAE only slightly (0.1289→0.1274 kcal/mol/Å) while reducing the seed-to-seed standard deviation roughly threefold. Therefore, its most reproducible benefits are a reduction in seed-to-seed variance and faster convergence (epochs to validation targets cut by 26–55%) rather than a large peak-accuracy gain, while energy accuracy is preserved (≈0.0355 kcal/mol). The choice of auxiliary target matters, as the hybrid target preserves force accuracy but interacts poorly with SCTA on the energy axis, whereas the physics target is the safer operating point. The auxiliary effect is scale-dependent: at the 100-epoch pilot configuration C used in the appendices, the auxiliary loss does not improve over SCTA alone (Appendix A.2), and the headline gain should accordingly be read as a paper-aligned-scale effect rather than a universal advantage at small scale. Limited cross-molecule probes on ethanol, uracil, and salicylic acid are reported only to delimit the scope of this finding: across all three, GotenNet + aux (physics) stays within a few percent of the reported GotenNet force MAE; however, at single-seed precision and without paired in-house baselines, this does not establish a molecule-independent claim. For the small ethanol effect in particular, the most plausible explanation is that its converged force MAE is already comparable to the spread of strong baselines on this molecule (NequIP, MACE, and Allegro all fall within ∼0.048–0.065 kcal/mol/Å per the GotenNet benchmark), leaving little room for any low-cost modification.

### Limitations and Future Directions

The present study covers one fully controlled molecule across three random seeds and three limited transfer probes, so the narrow 0.1280–0.1292 kcal/mol/Å single-seed force-MAE band among auxiliary-trained aspirin models should not be read as a definitive ranking. The ethanol, uracil, and salicylic acid comparisons use reported GotenNet numbers (five-split averages) rather than full paired multi-seed reproductions, and should be treated accordingly. Multi-seed validation on aspirin is now provided (Section 2.2), leaving multi-molecule validation across seeds as the most important remaining step. We evaluate models on held-out test configurations only, and do not assess long-horizon molecular-dynamics stability (energy drift, force-error tail behavior, or RMSD divergence under nanosecond-scale rollouts), which is increasingly used to gauge the practical utility of small MAE improvements. We regard this as the main limitation of our practical perspective: a converged-MAE difference as small as the 0.0023 kcal/mol/Å we report need not translate into a difference in trajectory stability, and an auxiliary loss that slightly lowers force MAE could help or hurt energy conservation over long rollouts. Therefore, establishing whether the auxiliary supervision delivers a genuine practical benefit would require NVE/NVT rollouts measuring energy drift and RMSD divergence, which we identify as the key practical followup enabled by the released checkpoints. Further work should test whether auxiliary geometric supervision transfers to newer no-CG or inner product-based backbones such as EST [35], Geodite [37], and MARA [38], whether SCTA benefits from force- and energy-specific readouts, and whether four-body or dihedral-aware attention improves the treatment of torsional geometry [20,57,58]. We release the code, configurations, checkpoints, and logs to support these followup studies.

## Figures and Tables

**Table 1:** Test set MAE on rMD17 aspirin, in kcal/mol for energy and kcal/mol/Å for force. The energy and force MAE are defined in Equations (17) and (18), respectively. Literature baselines are cited from the original references and converted to these units when necessary. Our rows use the GotenNet-aligned 950/50/1000 train/validation/test split [46] and are evaluated at the best-validation checkpoint under the paper-aligned protocol (Section 3.6). **Bold** indicates best per column. Analytic test set 95% CIs for the six in-house rows are reported in the discussion below.

Method	Energy MAE (kcal/mol)	Force MAE (kcal/mol/Å)
SchNet [48]	0.369	1.349
PaiNN [49]	0.159	0.339
NequIP [21]	0.053	0.196
Allegro [22]	0.053	0.168
MACE [23]	0.051	0.152
Graph-ACE [36]	0.039	0.141
GotenNet (reproduced)	**0.0353**	0.1303
SCTA (ours)	0.0360	0.1298
GotenNet + aux (hybrid)	0.0370	0.1292
GotenNet + aux (physics)	0.0357	**0.1280**
SCTA + aux (physics)	0.0397	0.1290
SCTA + aux (hybrid)	0.0538	0.1287

**Table 2:** Multi-seed test set comparison on rMD17 aspirin, reported as mean ± standard deviation over three random seeds (force in kcal/mol/Å, energy in kcal/mol).

Method	Force MAE	Energy MAE
GotenNet	0.1289±0.0018	0.0354±0.0003
GotenNet + aux (physics)	0.1274±0.0006	0.0355±0.0010

**Table 3:** Epochs to reach validation MAE thresholds on rMD17 aspirin at paper-aligned scale and the corresponding speedup relative to GotenNet. Here, SCTA + aux denotes SCTA combined with the hybrid graph-level auxiliary geometric target from Section 3.4.

	Epochs to Reach			Speedup vs. GotenNet		
Threshold	GotenNet	SCTA	SCTA + Aux	GotenNet	SCTA	SCTA + Aux
*Force MAE* (kcal/mol/Å)						
val_F ≤ 0.50	57	64	64	1.00×	0.89×	0.89×
val_F ≤ 0.30	179	156	132	1.00×	1.15×	1.36×
val_F ≤ 0.25	284	245	211	1.00×	1.16×	1.35×
val_F ≤ 0.20	323	337	295	1.00×	0.96×	1.09×
val_F ≤ 0.15	559	543	498	1.00×	1.03×	1.12×
val_F ≤ 0.13	917	987	859	1.00×	0.93×	1.07×
*Energy MAE* (kcal/mol)						
val_E ≤ 0.50	33	27	15	1.00×	1.22×	2.20×
val_E ≤ 0.20	96	79	76	1.00×	1.22×	1.26×
val_E ≤ 0.10	240	235	268	1.00×	1.02×	0.90×
val_E ≤ 0.05	464	638	448	1.00×	0.73×	1.04×
val_E ≤ 0.04	594	1077	742	1.00×	0.55×	0.80×

**Table 4:** Epochs-to-threshold for SCTA + aux (hybrid) measured against the strong GotenNet + aux (physics) baseline rather than vanilla GotenNet. The SCTA-specific advantage is sign-inconsistent across force thresholds, and should be interpreted cautiously at single-seed precision.

Threshold	GotenNet + Aux (Physics)	SCTA + Aux (Hybrid)	Ratio
*Force MAE* (kcal/mol/Å)			
val_F ≤ 0.50	59	64	0.92×
val_F ≤ 0.40	97	100	0.97×
val_F ≤ 0.30	175	132	1.33×
val_F ≤ 0.25	197	211	0.93×
val_F ≤ 0.20	330	295	1.12×
val_F ≤ 0.15	498	498	1.00×
*Energy MAE* (kcal/mol)			
val_E ≤ 0.50	27	15	1.80×
val_E ≤ 0.20	117	76	1.54×
val_E ≤ 0.10	190	268	0.71×

**Table 5:** Test set MAE on rMD17 aspirin at the best-validation checkpoint, with energy in kcal/mol and force in kcal/mol/Å. The rightmost column reports the validation-to-test inflation of energy MAE, (test_E−val_E)/val_E, where test_E is the test energy MAE at the best-validation checkpoint and val_E is the best (minimum) validation energy MAE reached during training. **Bold** indicates best per column.

Config	Test_E	Test_F	Val→Test E Gap
GotenNet	**0.0353**	0.1303	+20%
SCTA	0.0360	0.1298	** +1% **
GotenNet + aux (hybrid)	0.0370	0.1292	+34%
GotenNet + aux (physics)	0.0357	**0.1280**	+33%
SCTA + aux (physics)	0.0397	0.1290	+20%
SCTA + aux (hybrid)	0.0538	0.1287	+69%

**Table 6:** Limited rMD17 cross-molecule probes (energy in kcal/mol, force in kcal/mol/Å). “GotenNet (reported)” rows are five-split averages from [46]; “in-house” rows are single-seed single-split runs.

Molecule	Method	Energy MAE	Force MAE
ethanol	GotenNet (reported)	0.0071	0.0482
ethanol	GotenNet + aux (physics, this work)	0.00741	0.05022
ethanol	SCTA + aux (physics, this work)	0.00763	0.05186
uracil	GotenNet (reported)	0.0064	0.0417
uracil	GotenNet + aux (physics, this work)	0.00577	0.04248
salicylic acid	GotenNet (reported)	0.0141	0.0703
salicylic acid	GotenNet (in-house, paired)	0.01150	0.06885
salicylic acid	GotenNet + aux (physics, this work)	0.01356	0.07013

**Table 7:** Validation MAE on rMD17 aspirin under pilot configuration B of Table A4, comparing GotenNet with and without SCTA at $L_{\max}\in \left\{1,2\right\}$.

Config	$L_{\max}$	SCTA	Best Val_F	Best Val_E
A	2	–	1.474	3.217
B	1	–	2.092 (+42%)	3.188
C	1	yes	2.267 (+54%)	3.258
D	2	yes	**1.334** (−9.5%)	**1.521** (−53%)

## Data Availability

Data are contained within the article. Code, configurations, checkpoints, and training logs supporting the reported results are released by the authors to enable reproduction.

## Abbreviations

    The following abbreviations are used in this manuscript:

SCTA	Scalarization-Compatible Triplet Cross-Attention
GNN	Graph Neural Network
MLIP	Machine-Learned Interatomic Potential
MAE	Mean Absolute Error
DFT	Density Functional Theory

MD	Molecular Dynamics
CG	Clebsch–Gordan (tensor product)
GATA	Graph Attention Transformer Architecture (from GotenNet)
EQFF	Equivariant Feed-Forward (from GotenNet)
HTR	Hierarchical Tensor Refinement (from GotenNet)
RBF	Radial Basis Function
MLP	Multi-Layer Perceptron
EMA	Exponential Moving Average
PBE	Perdew–Burke–Ernzerhof (DFT functional)
rMD17	Revised MD17 molecular dynamics dataset

## Appendix A. Frame Projection vs. SCTA: Empirical Comparison and Algebraic Analysis

This appendix presents the frame projection control summarized in Section 2.8 in two parts. First, we report an empirical comparison at pilot configuration C (Table A4) between SCTA’s cos-angle scalar branch and a frame-projection alternative that projects equivariant tensor features onto a triplet-local frame. Second, we analyze algebraically what each branch’s scalar features actually expose, and prove two structural properties of the frame-projection features: (i) the diagonal projected pairwise scalar is exactly frame-independent under orthonormality, reducing to an ordinary tensor inner product in the original Cartesian coordinate system; and (ii) the only genuinely frame-dependent channel is parity-odd, and consequently mismatched to a scalar parity-even energy target on achiral molecules. The empirical comparison shows that the two branches achieve comparable validation MAE at pilot scale, so the algebraic analysis is read here as a design rationale rather than as an explanation for an empirical failure: SCTA’s cos-angle is a true O(3) scalar invariant of the triplet and does not require a frame construction, which is the relevant observation for the design choice made in the main text.

### Appendix A.1. Setup: Frame Construction

For a triplet (i,j,k) with *i* as the center, we construct an orthonormal frame $F={\left[{\hat{e}}_{1},{\hat{e}}_{2},{\hat{e}}_{3}\right]}^{⊤}$ by

(A1) $$\begin{array}{cc} {\hat{e}}_{1} & ={\hat{r}}_{ij}, \end{array}$$

(A2) $$\begin{array}{cc} {\hat{e}}_{2} & =\mathrm{GS}\left({\hat{r}}_{ik},{\hat{e}}_{1}\right)=\frac{{\hat{r}}_{ik}-\left({\hat{r}}_{ik}\cdot {\hat{e}}_{1}\right)\;{\hat{e}}_{1}}{∥{\hat{r}}_{ik}-\left({\hat{r}}_{ik}\cdot {\hat{e}}_{1}\right)\;{\hat{e}}_{1}∥}, \end{array}$$

(A3) $$\begin{array}{cc} {\hat{e}}_{3} & ={\hat{e}}_{1}\times {\hat{e}}_{2}, \end{array}$$

where ${\hat{r}}_{ij}$ and ${\hat{r}}_{ik}$ are unit vectors along the edges $r_{ij}$ and $r_{ik}$. The frame rotates equivariantly with the triplet under SO(3), while Gram–Schmidt ensures the orthonormality $F^{⊤}F=I$ (with ${\hat{e}}_{3}$ a pseudovector under parity).

We project the rank-1 tensor features $X_{j},X_{k},X_{i}\in R^{3\times D}$ onto the frame:

(A4) $$s_{j}=FX_{j},\;s_{k}=FX_{k},\;s_{i}=FX_{i}\;\in \;R^{3\times D}\;$$

and extract two types of scalar features:

(A5) $$\begin{array}{ccc} {\mathrm{feat}}_{jk}\left[c\right] & =\sum_{\alpha =1}^{3}s_{j}\left[\alpha ,c\right]\;s_{k}\left[\alpha ,c\right] & (\mathrm{diagonal}\;\mathrm{projected}\;\mathrm{pairwise}\;\mathrm{scalar}) \end{array}$$

(A6) $$\begin{array}{ccc} {\mathrm{feat}}_{i3}\left[c\right] & =s_{i}\left[3,c\right] & (\mathrm{handedness}-\mathrm{dependent}\;\mathrm{third}-\mathrm{frame}\;\mathrm{component}) \end{array}$$

which are concatenated to form a two-dimensional triplet feature. This feature feeds an attention module analogous to SCTA and uses the same zero-initialized LayerScale residual connection.

### Appendix A.2. Empirical Comparison at Pilot Configuration C

We compare four ways of attaching the frame projection branch with SCTA’s cos-angle branch and a vanilla GotenNet baseline under pilot configuration C (Table A4: $d_{ne}=16$, two interaction layers, $L_{\max}=2$, $N_{\mathrm{ang}}=8$, batch 32, 100 epochs, single seed 42, with bias-free linear layers in the frame branch). The frame-attachment variants are as follows: parallel to SCTA, fully replacing SCTA, and a detached-frame replacement.

**Table A1:** Empirical comparison of scalar three-body branches on rMD17 aspirin at pilot configuration C. Energy MAE in kcal/mol, force MAE in kcal/mol/Å; both are best-validation values over 100 epochs (single seed 42).

Method	Val *E* MAE	Val *F* MAE
GotenNet baseline	1.47	2.01
SCTA	1.24	1.85
SCTA + aux	1.31	1.93
Frame projection in parallel with SCTA	1.26	1.85
Frame projection replaces SCTA	**0.87**	**1.83**
Frame projection replaces SCTA (detached)	**0.87**	**1.81**

We highlight three points from this table. First, the three scalar three-body branches (SCTA’s cos-angle attention, frame projection in parallel with SCTA, and frame projection as a replacement for SCTA) achieve essentially indistinguishable force MAE at this scale (val *F*∈[1.81,1.85] kcal/mol/Å), all improving over the vanilla GotenNet baseline (2.01) by roughly 8–10%. Second, the frame projection replacement variants also noticeably lower the energy MAE (val *E*≈0.87 vs. 1.24 for SCTA) without corresponding force degradation, and detaching the frame from the autograd graph does not change this picture. Third, the auxiliary loss does not help at pilot scale (1.93 vs. 1.85 for SCTA alone); this is consistent with the paper-aligned ablation, where the auxiliary effect on aspirin is small and threshold-dependent even at full scale, as well as with the noise floor argument in Section 2.5 that the smaller and faster-trained pilot is even closer to its own error floor.

Therefore, the empirical comparison does not separate the two scalar three-body designs at pilot scale. This is consistent with the algebraic observations developed below: the diagonal projected channel ${\mathrm{feat}}_{jk}$ is information-equivalent to a tensor inner product that the GotenNet backbone already exposes via HTR (Observation A1), so a frame-projection branch does not introduce new geometric content beyond what the backbone provides. In addition, the remaining frame-dependent channel ${\mathrm{feat}}_{i3}$ is parity-odd (Observation A2), so a learned weighting of it is not constrained to align with a parity-even energy target on achiral data. Whether either channel becomes a fragile gradient sink at paper-aligned scale (Configuration A) is not tested here, and is left as a falsification target for future work. Therefore, the algebraic analysis below is presented as a design rationale for choosing the cos-angle branch of SCTA in the main text (a true O(3)-invariant three-body scalar that requires no frame construction) rather than as an explanation of a frame-projection failure that the present pilot-scale data would not support.

### Appendix A.3. Algebraic Interpretation

Two elementary observations characterize what the frame-projection branch actually exposes. The first is an exact identity that removes the explicit frame dependence, while the second is a symmetry mismatch between the remaining frame-dependent feature and a scalar energy target on achiral molecules.

**Observation** **A1** (frame collapse). *For any orthonormal triplet frame F (i.e., $F^{⊤}F=I$), the main pairwise scalar feature reduces identically to the tensor inner product in the original Cartesian coordinate system, independent of the choice of frame:*

(A7) $${\mathrm{feat}}_{jk}\left[c\right]\;=\;\sum_{\alpha =1}^{3}s_{j}\left[\alpha ,c\right]\;s_{k}\left[\alpha ,c\right]\;=\;\langle X_{j}\left[\cdot ,c\right],\;X_{k}\left[\cdot ,c\right]\rangle .\;$$

*Thus, the diagonal projected scalar does not encode the local frame itself; at most, it re-injects a pairwise tensor contraction already aligned with the type of scalar information used by the backbone’s HTR pathway*.

**Observation** **A2** (pseudoscalar channel). *The only genuinely frame-dependent component, ${\mathrm{feat}}_{i3}\left[c\right]=s_{i}\left[3,c\right]=X_{i}\left[\cdot ,c\right]\cdot \left({\hat{e}}_{1}\times {\hat{e}}_{2}\right),$ is a triple product of three vectors, odd under parity inversion, and consequently a *pseudoscalar*. It is sensitive to handedness rather than to an ordinary O(3)-invariant angle magnitude. For the achiral rMD17 molecules used here, this signed handedness channel is not expected to align robustly with the scalar energy target:*

(A8) $${\mathrm{feat}}_{i3}\left[c\right]\overset{P}{\to }-{\mathrm{feat}}_{i3}\left[c\right].$$

#### Consequence

Combining these two observations, the frame projection branch contributes the following:

- *D* feature dimensions from ${\mathrm{feat}}_{jk}$ with local-frame dependence that cancels exactly by $F^{⊤}F=I$.
- *D* feature dimensions from ${\mathrm{feat}}_{i3}$ that are parity-odd, and consequently poorly matched to the scalar parity-even energy target on achiral molecules.

In summary, the frame projection branch contributes representational degrees of freedom with explicit frame dependence that either cancels (${\mathrm{feat}}_{jk}$) or is parity-odd (${\mathrm{feat}}_{i3}$), rather than a frame-dependent yet parity-even three-body invariant of the kind that a scalar energy target on achiral molecules can exploit directly.

**Proof of Observation A1** **(Frame Collapse of the Pairwise Scalar Feature).** Let *F* = ${\left[{\hat{e}}_{1},{\hat{e}}_{2},{\hat{e}}_{3}\right]}^{⊤}$$\in R^{3\times 3}$ be the orthonormal triplet frame constructed in Appendix A.1. The projection of a rank-1 tensor feature $X_{j}\in R^{3\times D}$ onto the frame is $s_{j}=FX_{j}\in R^{3\times D}$, with entries $s_{j}\left[\alpha ,c\right]=\sum_{\beta }F\left[\alpha ,\beta \right]\;X_{j}\left[\beta ,c\right]$. Then, for each channel c∈{1,…,D},

(A9) $$\begin{array}{cc} {\mathrm{feat}}_{jk}\left[c\right]\; & =\sum_{\alpha =1}^{3}s_{j}\left[\alpha ,c\right]\;s_{k}\left[\alpha ,c\right]\\ \; & =\sum_{\alpha =1}^{3}\left(\sum_{\beta }F\left[\alpha ,\beta \right]\;X_{j}\left[\beta ,c\right]\right)\left(\sum_{\gamma }F\left[\alpha ,\gamma \right]\;X_{k}\left[\gamma ,c\right]\right)\\ \; & =\sum_{\beta ,\gamma }X_{j}\left[\beta ,c\right]\;X_{k}\left[\gamma ,c\right]\underset{={\left[F^{⊤}F\right]}_{\beta \gamma }=\delta_{\beta \gamma }}{\underset{︸}{\sum_{\alpha =1}^{3}F\left[\alpha ,\beta \right]\;F\left[\alpha ,\gamma \right]}}\\ \; & =\sum_{\beta }X_{j}\left[\beta ,c\right]\;X_{k}\left[\beta ,c\right]\\ \; & =\langle X_{j}\left[\cdot ,c\right],\;X_{k}\left[\cdot ,c\right]\rangle , \end{array}$$

where we invoke the orthonormality $F^{⊤}F=I$ (equivalent to $\sum_{\alpha }F\left[\alpha ,\beta \right]\;F\left[\alpha ,\gamma \right]=\delta_{\beta \gamma }$) in the third line. The final expression depends only on the tensor features $X_{j},X_{k}$ in the original Cartesian coordinate system, and is manifestly *independent of the choice of frame F*. Consequently, ${\mathrm{feat}}_{jk}$ coincides with a pairwise tensor inner product rather than a genuinely frame-dependent triplet descriptor.    □

**Proof of Observation A2** **(Parity of the Third-Frame Component).** The remaining frame-dependent feature is the third-row component of $s_{i}$:

(A10) $${\mathrm{feat}}_{i3}\left[c\right]=s_{i}\left[3,c\right]=\sum_{\beta }F\left[3,\beta \right]\;X_{i}\left[\beta ,c\right]=X_{i}\left[\cdot ,c\right]\cdot {\hat{e}}_{3}=X_{i}\left[\cdot ,c\right]\cdot \left({\hat{e}}_{1}\times {\hat{e}}_{2}\right).\;$$

Substituting ${\hat{e}}_{1}={\hat{r}}_{ij}$ and the Gram–Schmidt expression for ${\hat{e}}_{2}$ into Equation (A10) and using the invariance of $\sin\theta_{jik}$ under rotation, we equivalently obtain

(A11) $${\mathrm{feat}}_{i3}\left[c\right]\;=\;\frac{X_{i}\left[\cdot ,c\right]\cdot \left({\hat{r}}_{ij}\times {\hat{r}}_{ik}\right)}{\sin\theta_{jik}},\;$$

which is a normalized triple product of the three vectors ${\hat{r}}_{ij},{\hat{r}}_{ik},X_{i}\left[\cdot ,c\right]$. Under spatial inversion r↦−r (parity), polar vectors such as ${\hat{r}}_{ij}$, ${\hat{r}}_{ik}$, and an equivariant rank-1 feature $X_{i}\left[\cdot ,c\right]$ change sign, while the cross product ${\hat{r}}_{ij}\times {\hat{r}}_{ik}$ is an axial vector and does not change sign. Therefore, the dot product of $X_{i}\left[\cdot ,c\right]$ with this axial vector changes sign:

(A12) $${\mathrm{feat}}_{i3}\left[c\right]\;\overset{P}{\to }\;-{\mathrm{feat}}_{i3}\left[c\right],$$

identifying ${\mathrm{feat}}_{i3}$ as a pseudoscalar under O(3). A pseudoscalar can be informative for chiral targets or for tasks that explicitly require handedness; however, in the present scalar energy/force benchmark on achiral molecules, this channel is not an ordinary invariant geometric descriptor like a distance or angle magnitude, making it a fragile scalarization choice for the auxiliary triplet branch.    □

### Appendix A.4. SCTA’s Cos-Angle Design as the Principled Non-Degenerate Alternative

The above analysis shows that frame projection’s two scalar outputs have specific structural limitations: (i) the main pairwise feature is exactly frame-independent under orthonormality, so it carries no information beyond an ordinary tensor inner product, and (ii) the remaining frame-dependent feature is parity-odd, so it is not an O(3) scalar of the kind that a parity-even energy target on achiral molecules can exploit directly. Although the empirical comparison at pilot scale (Appendix A.2) does not separate frame projection from SCTA, these structural properties motivate SCTA’s cos-angle design as the more principled scalar three-body choice, since it avoids both issues by construction.

#### Appendix A.4.1. SCTA Avoids the Orthonormality Collapse

SCTA’s triplet feature is $\cos\theta_{jik}={\hat{r}}_{ij}\cdot {\hat{r}}_{ik}$, a scalar function of the two edge unit vectors at the center. Unlike frame projection, this quantity does not involve any frame matrix *F*, meaning that no Gram–Schmidt orthogonalization or $F^{⊤}F=I$ identity applies. The cosine $\cos\theta_{jik}$ is a *genuine three-body invariant*: although it is algebraically related to the three distances of a triangle by the law of cosines, SCTA exposes this relation explicitly for each ordered neighbor pair (j,k) around center atom *i*. Therefore, the attention branch receives an angle-resolved triplet descriptor rather than a frame-projected tensor inner product for which the frame dependence cancels.

#### Appendix A.4.2. SCTA Avoids the Pseudoscalar Pitfall

The cosine cosθ is a true scalar under O(3), that is, it is invariant under both rotations and reflections. As there is no pseudoscalar component in SCTA’s triplet feature, there is no parity-odd handedness channel. The same SCTA attention computation applies identically to chiral, achiral, and planar molecules, and is not subject to the parity mismatch of the third-frame component.

#### Appendix A.4.3. SCTA’s Gaussian Angular Basis Contributes Independent Feature Dimensions

The Gaussian projection $\phi_{n}\left(\cos\theta \right)=\exp\left(-\gamma {\left(\cos\theta -\mu_{n}\right)}^{2}\right)$ with $n=1,\ldots ,N_{\mathrm{ang}}$ maps the single scalar cosθ into a localized $N_{\mathrm{ang}}$-dimensional angular basis. Therefore, the resulting triplet attention score $q_{i}⊙k_{j}⊙k_{k}⊙\psi \left(\theta_{jik}\right)$ combines *three types of scalar information:* pairwise features via q,k, three-body via cosθ, and nonlinear angular resolution via ψ.

#### Appendix A.4.4. Implication

In the design space of scalar-first non-Clebsch–Gordan three-body attention on achiral force-field tasks, SCTA’s cos-angle basis is the cleaner nondegenerate choice: it is neither collapsed by an orthonormal-frame identity (unlike ${\mathrm{feat}}_{jk}$), nor is it parity-odd (unlike ${\mathrm{feat}}_{i3}$). This places SCTA alongside scalar-first three-body methods such as DimeNet [59], GemNet [20], SphereNet [57], and ComENet [58], all of which rely on angle invariants rather than frame projections as their core geometric primitive. Thus, the empirical failure of the frame control supports the design choice made in SCTA, namely, that scalar attention should operate on explicit angle invariants rather than on diagonal frame-projected tensor inner products for the rMD17-class scalar energy/force tasks studied here.

### Appendix A.5. Remark: When Frame Projection Does Contribute Information

The two observations above identify the boundary of the negative result rather than generally invalidating frame-based designs. First, Observation A1 applies to the diagonal contraction over α together with $F^{⊤}F=I$; off-diagonal projected products $T_{jk}\left[\alpha ,\beta \right]=s_{j}\left[\alpha ,c\right]\;s_{k}\left[\beta ,c\right]$ with α≠β transform non-trivially under the frame, and can retain frame-dependent information. Such off-diagonal features may be useful for tensor-target tasks such as molecular polarizability tensor and NMR shielding tensor prediction. Second, the relevance of Observation A2 depends on the data and task. On chiral datasets (e.g., amino acids or sugar conformers), a pseudoscalar channel can correlate with handedness and help to distinguish enantiomers. Thus, our negative result on rMD17 states a *boundary condition* on the scalar frame projection design tested here, rather than a universal invalidation.

## Appendix B. Additional Ablations and Sensitivity Studies

### Appendix B.1. Hyperparameter Sensitivity

To verify that the pilot behavior of the SCTA + auxiliary loss design is not an artifact of a particular hyperparameter choice, we sweep its three main knobs at the reduced pilot configuration ($d_{ne}=32$, three interaction layers, aspirin, 100 epochs, seed 42). Sweeps are performed one dimension at a time, fixing the remaining knobs at the values used in the main experiments.

The $N_{\mathrm{ang}}$ sweep (rows 2–3) shows that the choice of eight Gaussian angular basis centers is not a critical hyperparameter: $N_{\mathrm{ang}}=4$ produces a marginally lower val_F (1.83) and $N_{\mathrm{ang}}=16$ a marginally higher one (2.03), so the full $N_{\mathrm{ang}}\in \left\{4,8,16\right\}$ range spans about 10% around the reference. The auxiliary weight $w_{A}$ sweep (rows 4–5) is essentially flat: $w_{A}=0.01$, 0.02, and 0.05 all give val_F within 1% of one another. The annealing schedule sweep (row 6) is also essentially flat: a 50-epoch linear anneal produces val_F 1.94 vs. 1.93 for the constant-weight reference. Together, these probes indicate that SCTA + aux is not delicately tuned at the pilot scale, with qualitative behavior that is robust to all three main knobs.

**Table A2:** Hyperparameter sensitivity on rMD17 aspirin (pilot configuration C of Table A4). Each row is a complete experimental setting; the first (bold) row is the reference used in the main results, and the subsequent rows each perturb a single hyperparameter.

$N_{\mathrm{ang}}$	$w_{A}$	$T_{\mathrm{Anneal}}$	Best val_F	Best val_E
**8**	**0.02**	**off**	**1.93**	**1.21**
4	0.02	off	1.83	1.15
16	0.02	off	2.03	1.21
8	0.01	off	1.94	1.19
8	0.05	off	1.93	1.20
8	0.02	50 ep	1.94	1.22

### Appendix B.2. Bond-Cutoff Sensitivity of the Auxiliary Target

The auxiliary geometric loss uses a fixed 1.8 Å bonded-distance cutoff to detect chemical bonds (Section 3.4). To verify that this threshold is not a tuned hyperparameter, we ran a small ablation at pilot configuration C with GotenNet + aux (physics) and three cutoff values: 1.6 Å (tight, excludes most C–O/C–C bonds in extended conformations); 1.8 Å (reference, captures all typical single bonds); and 2.0 Å (loose, includes long-range contacts).

**Table A3:** Bond-cutoff sensitivity for the physics auxiliary target at pilot configuration C (GotenNet + aux (physics), seed 42, 100 epochs). Best validation energy and force MAE.

Bond Cutoff (Å)	Best Val_F	Best Val_E	Relative to 1.8 Å
1.6 (tight)	2.036	1.376	+0.9% F, +5.9% E
**1.8 (reference)**	**2.018**	**1.299**	0% (reference)
2.0 (loose)	2.040	1.351	+1.1% F, +4.0% E

The reference 1.8 Å value is the best of the three on both axes, with a force-MAE spread of about 1% and an energy-MAE spread of about 6% across the sweep; therefore, the threshold is not a delicately tuned hyperparameter for rMD17 small organic molecules, as tightening it slightly degrades the auxiliary supervision (the bonded subgraph misses real bonds at thermal extension) and loosening it slightly worsens energy MAE without changing the qualitative behavior (more non-bonded contacts enter the bond set). This robustness check supports leaving the cutoff as a fixed hyperparameter at 1.8 Å in the main experiments rather than tuning it per molecule.

### Appendix B.3. Full Experimental Configurations

Table A4 summarizes the three distinct experimental configurations used across the paper. Configuration A is used for Section 2.1 (main results) and Section 2.3 (sample-efficiency analysis); Configuration B is used for the tensor-feature complementarity ablation in Section 2.7; and Configuration C is used for the frame projection ablation in Appendix A and hyperparameter sensitivity studies in Appendix B.1.

### Appendix B.4. Negative Architectural Variants Not Included in the Main Text

During the development of SCTA, we evaluated several architectural variants that failed to improve over the SCTA baseline at the pilot configuration (C). For completeness, we briefly summarize them here; detailed results are available in the accompanying code repository.

- **Non-negative gate (SCTA-N).** Replacing the zero-initialized LayerScale λ with a softplus-gated non-negative scalar yielded val_F =1.45 vs. SCTA’s 1.33. Therefore, negative λ channels in the converged SCTA model are a *feature* (allowing the triplet message to subtract from selected scalar channels), not a numerical artifact to be constrained away.
- **X-norm augmented query (SCTA-R).** Concatenating per-degree steerable-feature norms $∥{\tilde{X}}_{i}^{\left(l\right)}{∥}_{2}$ to the scalar query input before the $W_{q}$ projection (inspired by GotenNet’s HTR-style scalar readout) degraded val_F by ∼10%. Diagnostic analysis attributes the failure to the near-zero $\tilde{X}$ magnitude in the first few layers, for which a single linear projection of $\left[h,∥\tilde{X}∥\right]$ is ill-conditioned.
- **Controller-mode SCTA.** Rerouting the SCTA output to modulate the equivariant feature *X* (rather than adding to the scalar *h*) through a gated multiplicative update produced a range of val_F between 1.38 and 1.61 across five gate-initialization variants, all worse than the SCTA baseline 1.33. The regime at $d_{ne}=32,n_{\mathrm{layer}}=3$ is too shallow for the optimizer to learn non-trivial gate values within 100 epochs, causing the extra parameters to behave as noise.
- **Edge-anchored triplet bias (EATB).** Moving the SCTA aggregation from node-centered to edge-anchored attention, with the result injected into the edge state $t_{ij}$ pre-GATA, yielded val_F =1.40 at the pilot configuration. The non-residual injection appears to interfere with GATA’s learning dynamics under short training budgets.

**Table A4:** Summary of experimental configurations. All runs use AdamW with weight decay 0, ReduceLROnPlateau schedule (patience 30, factor 0.8), and EMA decay 0.9 for validation metric computation.

	A (Paper-Aligned)	B ($L_{\max}$ Ablation)	C (Pilot)
Hidden dimension *D*	192	32	16
Interaction layers	16	3	2
$L_{\max}$ (steerable)	2	{1,2}	2
Cutoff radius	5.0 Å	5.0 Å	5.0 Å
Radial basis size $N_{\mathrm{rbf}}$	32	32	32
Angular basis size $N_{\mathrm{ang}}$	8	8	8
Training epochs	3000	100	100
Learning rate	$2\times 10^{-4}$	$2\times 10^{-4}$	$2\times 10^{-4}$
Batch size	4	32	32

These negative results strengthen rather than weaken the case for the specific design chosen in the main text. At the pilot configuration, SCTA in its natural form (zero-initialized LayerScale residual on *h*, cos-angle attention, center-node aggregation) is the only variant in the explored space that improves over the GotenNet baseline.

### Appendix B.5. Pathway-Level Ablation of HTR: A Hook for Architectural Simplification

The ablation in Section 2.7 of the main text shows that SCTA does *not* substitute for the full $L_{\max}=2$ steerable-feature channel. Here, we examine a more fine-grained question: does SCTA overlap with a *specific sub-pathway* of HTR, namely, the inner product $\langle {\tilde{EQ}}_{i}^{\left(1\right)},{\tilde{EK}}_{j}^{\left(1\right)}\rangle$ for which the scalar (trace) part corresponds to cosθ—the same angular information that SCTA encodes explicitly? We implement two additional pilot variants at configuration C of Table A4: (i) **Stage 1**, consisting of vanilla GotenNet with the l=1⊗l=1 term removed from the HTR edge-weight sum (all other HTR terms intact); and (ii) **Stage 2**, consisting of the Stage 1 backbone augmented with the SCTA residual branch. Results are compared with the four configurations from Section 2.7 in Table A5.

**Table A5:** HTR pathway ablation on rMD17 aspirin (pilot configuration C of Table A4). Stage 1 removes the l=1⊗l=1 contribution from HTR’s edge-weight sum while keeping the $L_{\max}=2$ steerable features intact; Stage 2 further adds the SCTA residual branch.

Config	Best Val_F	Best Val_E
A. GotenNet full ($L_{\max}=2$)	1.474	3.217
Stage 1. GotenNet − HTR(l=1⊗l=1)	1.475 (+0.1%)	1.863
D. GotenNet + SCTA (full HTR)	**1.334** (−9.5%)	1.521
Stage 2. GotenNet + SCTA − HTR(l=1⊗l=1)	1.376 (−6.7%)	**1.316**

We highlight three points from this table.

**(i) The l=1⊗l=1 sub-pathway of HTR is nearly redundant at this configuration.** Removing it (A → Stage 1) costs only 0.1% on val_F and substantially improves val_E (3.22→1.86). This is unexpected; the specific angular information encoded as $\langle {\tilde{EQ}}_{i}^{\left(1\right)},{\tilde{EK}}_{j}^{\left(1\right)}\rangle$ appears to interfere with the scalar readout used for energy prediction while contributing little to force quality when $L_{\max}=2$ features remain available through the other HTR terms.

**(ii) Combining HTR l=1 removal with SCTA yields the lowest energy MAE observed.** Stage 2 reaches val_E =1.316, the best of all five configurations, while its val_F of 1.376 is within 3.1% of the full-HTR + SCTA configuration D. The 6.7% val_F gain over the vanilla baseline combined with the val_E improvement makes Stage 2 a viable but imperfect simplification of the full GotenNet + SCTA architecture.

**(iii) SCTA does not fully replace the l=1⊗l=1 scalar channel of HTR.** If the replacement were exact, Stage 2 val_F would match the 1.334 of configuration D; instead, it is 1.376, a 3.1% residual gap that points to a small but non-trivial synergy between the explicit cosθ attention in SCTA and the implicit l=1⊗l=1 encoding in HTR. Whether this gap persists, closes, or reverses at paper-aligned depth ($n_{l}=16$) is an open question.

Therefore, we leave partial pathway-level replacement to future work. A cleanly simplified variant in the form of GotenNet with the l=1⊗l=1 HTR term removed and replaced by SCTA and trained at paper-aligned scale for 3000 epochs would either match the full model (supporting replacement) or reveal the source of the 3.1% gap (for example, interactions between the pathway-sum structure of HTR and the per-channel attention in SCTA). Either outcome would tighten the scalar-first three-body design space documented in this paper.

### Appendix B.6. Conformational Variance of the Auxiliary Target

Section 2.5 attributes the small or absent auxiliary effect on ethanol to the low absolute error scale rather than to a degenerate (near-constant) supervision signal. To support this, we compute the physics-style auxiliary target $\left[\;\bar{\left|\cos\tau \right|},\;b_{\min},\;\sigma_{b}^{\mathrm{node}}\;\right]$ on all 950 training configurations of each molecule using the same code path as the trained models (neighbor graph at 5 Å, bond cutoff 1.8 Å). We report the per-component mean and coefficient of variation (CV, the conformation-to-conformation standard deviation divided by the mean) in Table A6.

Two points emerge from this table. First, ethanol is not a degenerate case: the CV of every component is comparable to or larger than the corresponding value for aspirin (e.g., $\bar{\left|\cos\tau \right|}$ CV 4.2% vs. 4.0%; $\sigma_{b}^{\mathrm{node}}$ CV 25.6% vs. 15.6%). Therefore, the auxiliary target carries usable conformational signal on ethanol, and the small auxiliary effect here is better explained by the low absolute error scale (Section 2.5) than by a near-constant supervision target. Second, uracil has the lowest target variance of the four molecules ($\bar{\left|\cos\tau \right|}$ CV 1.9%, $b_{\min}$ CV 2.9%), consistent with its rigid aromatic ring; this matches the observation that its training auxiliary loss decreases only mildly over the run, in contrast to the much larger relative decrease seen on more flexible molecules.

**Table A6:** Conformational statistics of the physics auxiliary target on the 950-configuration training split of each rMD17 molecule. We report the mean and the coefficient of variation (CV%) for each of the three target components. A higher CV indicates a target that varies more across conformations, meaning that it carries more usable supervision signal.

Molecule	Atoms	$\bar{\left|\cos\tau \right|}$ (Mean/CV%)	$b_{\min}$ (Mean/CV%)	$\sigma_{b}^{\mathrm{node}}$ (Mean/CV%)
ethanol	9	0.584 / 4.2	0.980 / 3.5	0.190 / 25.6
uracil	12	0.966 / 1.9	1.005 / 2.9	0.071 / 7.7
salicylic acid	16	0.977 / 1.1	0.971 / 3.0	0.095 / 21.7
aspirin	21	0.833 / 4.0	0.986 / 3.2	0.100 / 15.6

To understand the energy-axis instability of the *hybrid* target under SCTA (Section 2.4), we also compute the three *hybrid* components $\left[\;\bar{\cos\theta_{G}},\;\bar{\cos\tau_{G}},\;\mathrm{std}\left(\cos\tau_{G}\right)\;\right]$ on 3000 aspirin training configurations through the same code path (Table A7). The signed dihedral mean $\bar{\cos\tau_{G}}$ stands out in scale. Its magnitude is small (≈−0.04), roughly an order of magnitude below the other two components ($\bar{\cos\theta_{G}}\approx -0.33$ and $\mathrm{std}(\cos\tau_{G})\approx 0.87$), and it is tightly concentrated near but not at zero (every configuration falls within ±0.1). It is small not through an exact symmetry (a reflection maps τ→−τ, under which cosτ is unchanged) but because the signed cosines of the many bonded dihedrals largely cancel in the graph mean. However, this small magnitude does not make it a constant or a pure-noise target; it retains a conformation-to-conformation CV of ≈45%, which is comparable to the physics-target components in Table A6. Therefore, the hybrid instability under SCTA is better attributed to this single component being poorly scaled relative to the rest of the target vector than to a degenerate or noise-like signal. The *physics* target replaces it with the chirality-insensitive magnitude $\bar{|\cos\tau_{G}|}\approx 0.83$, which both restores a comparable scale and removes the instability.

**Table A7:** Conformational statistics of the three *hybrid* auxiliary-target components on the aspirin training configurations (same 5 Å neighbor graph and 1.8 Å bond cutoff as Table A6). The signed dihedral mean $\bar{\cos\tau_{G}}$ is an order of magnitude smaller in mean than the other two components, yet retains a high CV.

Hybrid Component	Mean	Std	CV%
$\bar{\cos\theta_{G}}$	−0.328	0.075	22.9
$\bar{\cos\tau_{G}}$	−0.036	0.016	44.8
$\mathrm{std}(\cos\tau_{G})$	+0.869	0.029	3.3

### Appendix B.7. Localized vs. Graph-Level Auxiliary Supervision

The graph-level auxiliary target of Section 3.4 collapses a molecule’s bond-angle and dihedral distribution to a few scalar moments, meaning that it supervises only aggregate statistics and cannot indicate where in the molecule a geometric error occurs. Several finer-grained and more localized alternatives are possible: (i) *per-bond or per-triplet regression*, attaching an edge-level or triplet-level head that predicts each bonded distance, bond angle $\cos\theta_{jik}$, or dihedral cosτ directly, i.e., one supervised value per local geometric element rather than one per graph; (ii) *distribution matching*, matching the full empirical histogram of bond angles and dihedrals (for example through a binned or sliced-Wasserstein loss) instead of its first two moments, which preserves multimodal torsional structure that a mean and standard deviation cannot; and (iii) *per-atom local descriptors* supervising a rotation-invariant summary of each atom’s local environment by interpolating between the per-triplet and graph-level extremes. Each of these would in principle provide a denser geometric gradient localized to the offending substructure.

However, these schemes are not obvious improvements. All three reintroduce a dependence on an explicit bond or triplet partition, which is itself brittle under thermal sampling. Covalent bonds stretch in finite-temperature configurations, so a per-bond or per-triplet label can become noisy or undefined in exactly the distorted geometries where geometric supervision should matter most; this is the same effect that motivates the conservative 1.8 Å bonded cutoff of Section 3.4. More importantly, the geometric target is a deterministic function of the input coordinates, and is computed under torch.no_grad; therefore, it carries no information beyond the network’s own input, and its value lies in acting as a weak prior on the scalar representation rather than in providing new physical signal. Making the target finer-grained converts this gentle low-dimensional prior into a high-dimensional input-redundant regression task that competes with the energy–force objective for capacity and can be satisfied by re-encoding the input geometry rather than by improving the potential. The energy-axis instability we already observe for the signed-dihedral hybrid target (Section 2.4) is a small-scale warning that a more aggressive geometric target could interfere with the prediction targets.

Finally, the distributional framing of scheme (ii) adds little at the single-configuration level. For one molecular configuration, the angle/dihedral “distribution” is simply the finite set of its triplet values, so per-configuration distribution matching reduces to per-triplet regression up to ordering while also inheriting the practical fragility of histogram or optimal-transport losses (bin-width hyperparameters, vanishing or relaxation-dependent gradients, and projection variance). For these reasons, we regard localized supervision as a promising but not obviously superior direction. The coarseness of the graph-level loss is partly intentional, as it yields a partition-free and low-cost regularizer that already shifts the converged force MAE at essentially zero cost. A controlled comparison of localized against graph-level supervision, together with the loss weighting and stability safeguards that such finer targets would require, is left to future work.
